# Artificial neural network modeling of engineering properties and microbial stability of watermelon juice preserved with sustainable natural additives

**DOI:** 10.3389/fmicb.2026.1977402

**Published:** 2026-10-09

**Authors:** Rowida Allily, Ekruyota Godwin, Alanood A. Alfaleh, Ebtihaj O. Alnasri, Maha Hijazi, Uguru Hilary, Edafiadhe Emozino, Nimah Alnemari, Rokayya Sami, Mahmoud Helal, Amera N. Alqahtani, Awatif Almehmadi, Ola Abu Ali, Sarah Alharthi, Ahmad F. Alhomodi

**Affiliations:** 1 Department of Food and Nutrition, Faculty of Human Sciences and Design, King Abdulaziz University, Jeddah, Saudi Arabia; 2 Department of Cyber Security, Southern Delta University, Ozoro, Nigeria; 3 Department of Agricultural Engineering, Southern Delta University, Ozoro, Nigeria; 4 Department of Mechanical Engineering, Southern Delta University, Ozoro, Nigeria; 5 Department of Food Science and Nutrition, College of Sciences, Taif University, Taif, Saudi Arabia; 6 Department of Mechanical Engineering, Faculty of Engineering, Taif University, Taif, Saudi Arabia; 7 Department of Biological Sciences, College of Science, University of Jeddah, Jeddah, Saudi Arabia; 8 Department of Clinical Nutrition, Faculty of Applied Medical Sciences, Umm Al-Qura University, Makkah, Saudi Arabia; 9 Department of Chemistry, College of Science, Taif University, Taif, Saudi Arabia; 10 Research Center of Basic Sciences, Engineering and High Altitude, Taif University, Taif, Saudi Arabia; 11 Department of Biology, College of Science and Arts, Najran University, Najran, Saudi Arabia

**Keywords:** antioxidant, artificial neural network, microbial stability, phytochemicals, projection

## Abstract

Food quality preservation remains a major challenge globally, especially for highly labile fruit juices. This study was conducted to predict the engineering and microbial stability of bio-fortified watermelon juice, using linear regression analysis and an artificial neural network (ANN). In this study, watermelon juice was treated with the synthetic additive sodium benzoate and natural (sustainable) additives, including clove seed extract, cinnamon extract, bay leaf extract, ginger rhizome extract, honey, and lime juice. The results revealed that the natural additives significantly improved, the engineering properties, antioxidant ability, and antimicrobial potential of the treated juice samples (*p* ≤ 0.05), with some of the multi-component hybridized treatment formulations (particularly Treatment T13), performing better than the synthetic additive. Particularly, the negative control unit exhibited the poorest DPPH, TPC, TFC, FRAP, Vitamin C, and antimicrobial values, while the T13 sample recorded the maximum values of 91.30% DPPH, 191.86 mg GAE/100 mL juice, 77.31 mg QE/100 mL juice, 10.37 μmol Fe^2+^/mL, and 17.82 mg/100 mL, respectively. Also, the T13 sample recorded the lowest bacterial and fungal counts of 3.29 ×10^4^ and 2.17 ×10^3^ CFU/mL after 10 storage days, respectively. The results of the linear regression analysis, microbial inhibition efficiency, and index comparison further affirmed that the sustainable additives can substantially improve the antioxidant and antimicrobial mechanisms of the treatment. The linear regression modeling elucidated the associations between treatment and storage time and the evaluated quality attributes; conversely, the ANN approach acted as a supplementary nonlinear strategy for anticipating selected bioprocess and microbial behavior. Basically, the findings of this research have highlighted that, combined natural additives and computer programming, have potential to improve antioxidant effects, microbial stability, and targeted biophysical characteristics of watermelon juice. These findings suggest preservation capacity without demonstrating definitive clinical or epidemiological benefits.

## Introduction

Food safety and security are important aspects in human and capital development. These factors play essential parts, in safeguarding humans’ adequate access to affordable and nutritious food; hence, minimizing the occurrences of food poisoning and economic loss (Onyeaka et al., 2021; Mengistu et al., 2021; Wani et al., 2024). Anthropogenic factors that facilitate food spoilage and consequent foodborne aliments include: poor food preparation practices and cross contamination by handlers. Pathogenic microorganisms have the prospect of high survivability and performance in high moisture medium, warm environment, as well as in the presence of sugars, lower acidity, milk and proteins (Kochhar et al., 2022; Mohamad et al., 2022). Microorganisms–spoilage agents and pathogens, are responsible for about 30% of food wastage annually as well as food poisoning, resulting in an estimated US$ 310 billion loss associated with food spoilage, which is a major threat to food security and human health (Karanth et al., 2023; Pereira et al., 2026; WHO, 2024). Recently, computer science has become progressively incorporated into food science, through machine learning techniques that offer robust prognostic models for smart food systems.

Fruit juice is among the most widely consumed food products globally. This is primarily due sustainability of their raw materials, preparation simplicity, high nutritional and medicinal levels. Fruit juice is rich minerals, antioxidants and other essential bioactive compounds (Mohamad et al., 2022; Lima et al., 2026). Therefore, it has many nutritional and pharmaceutical values such as: antimicrobial properties, anti-inflammatory properties, weight management effects, and cardiovascular management. Though these anti-microbial and antioxidants properties are highly dependent on the plant genotype, cropping and environmental conditions, pre-harvest and post-harvest management, and processing technique (Adewoyin, 2023; Tao et al., 2026).

Among the numerous fruit juices, watermelon (*Citrullus lanatus*) juice is one of the most commonly consumed due to its high health benefits. It has an extraordinary hydration ability and nutritional profile (Ridwan et al., 2018; Mohamad et al., 2022; Lima et al., 2026). Studies have shown that watermelon juice is rich in basic phytochemicals, with excellent amount of amino acid citrulline, lycopene, vitamin C, and antioxidant potentials. These compounds are beneficial to health, such as in the prevention and management of cardiovascular disease, obesity, diabetes, and cancer (Tarazona-Díaz et al., 2010; Manivannan et al., 2020; Narayanan et al., 2024). The L-citrulline content of watermelon juice facilitates the relaxation and dilation of blood vessels, thereby preventing arterial stiffness and consequently lowering blood pressure (Luo et al., 2025). Watermelon juice is prone to drastic alterations in its physiochemical and engineering properties during storage, especially at high temperatures, due to microbial degradation and oxidation. Studies have revealed that watermelon fruit juice has a very high water activity (about 98%) and low acidity, with a pH of about 6.0. Therefore, it is highly prone to microbial growth and enzymatic deterioration (Hu et al., 2022; Lee et al., 2021; Lima et al., 2026).

Poorly processed fruits juices are susceptible to microbial infestation, which is exacerbated by poor fruit quality, sanitary condition, packaging, and storage operations. Microbial activities compromise the nutritional values and antioxidant properties of fruit juice, resulting in the accumulation of pathogenic and toxic biocompounds, which may pose public health risks and decline in profitability (Karanth et al., 2023; Tenea et al., 2023; Mafe et al., 2024; FDA, 2021). According to the World Health Organization (WHO), about 500,000 people die annually from food poisoning, and US$ 110 billion is lost yearly due to cases associated with food poisoning. This has severely impeded socioeconomic development, national economies, tourism, and capital development (WHO, 2024). Pathogenic microorganisms such as *Salmonella, Vibrio cholerae, Campylobacter, Listeria*, and enterohaemorrhagic *Escherichia coli*, are some of the most common pathogens found in fruit juices that have fatal health consequences (WHO, 2024).

For decades, synthetic antimicrobial preservatives have been widely used to prolong the microbial durability of fruit juices. These inorganic compounds, such as sodium benzoate, potassium sorbate, sodium metabisulphite (or sulfite), calcium propionate, and potassium nitrate, have the potential to inhibit the growth of pathogenic microorganisms, impair oxidation, prevent fermentation, and interrupt of the formation of toxic compounds (Musyoka et al., 2018; Chen and Zhong, 2018; Lee et al., 2021). Elevated concerns about the ecological impact, regulatory constraints, and potential toxicity related to the overuse or improper application of certain artificial preservatives have heightened interest in naturally sourced preservation methods. This is because excessive usage (prolonged exposure) of artificial preservatives, has been attributed to cancer, DNA damage, gastrointestinal disorders, kidney failure, and liver problems (Olas et al., 2021; Walczak-Nowicka and Herbet, 2022; Besedin et al., 2024; Pinto et al., 2025; Jayaraman et al., 2025).

Public health concerns, linked to synthetic food preservatives have resulted in a paramount quest, for more sustainable and health-friendly food additives. This has resulted in the development and utilization of natural preservatives, mostly plant derivatives, which are also more sustainable (Quinto et al., 2019; Mafe and Büsselberg, 2025; Albishi et al., 2026). Plant derivatives contain a wide range of organic compounds, though their numbers and concentrations vary widely, mainly depending on the plant genotype. These bioactive (organic) compounds play multiple roles in food processing and other engineering applications, owing to their ability to enhance the food’s nutritional quality, as well as its antioxidant, antimicrobial, and anti-inflammatory properties (Petcu et al., 2023; Maddaloni et al., 2025; Zhu and Yin, 2025).

Most convectional fruit juice quality evaluation techniques primarily relied on laboratory analysis, which is costly and time consuming in most cases. These bottle-neck technicalities tend to lead to compromise results, resulting to either food wastage or/and possible health implications (Sionek et al., 2024). Machine learning, artificial neural networks (ANN) and artificial intelligence (AI) are crucial instruments, used in monitoring food quality and public safety. These approaches use variables, programmes and algorithms to monitor the food quality; thereby minimizing the occurrence of foodborne diseases and spoilage (Maoto et al., 2024; Dehdashti et al., 2026; Yusufoğlu et al., 2026). AI-based models can substantially reduce laboratory work, and ensure high projected accuracy, making them vital tools for investigating complex experiments and maintaining quality control (Warade, 2025). Predictive models that use machine learning, such as ANNs, help detect early signs of food system deterioration. They employ organized tactics, to classify the major underlying causative factors and identify possible remedies, before the situation results in significant quality and public health problems (Shehzad, 2025).

Although several studies have explored sustainable materials as food preservatives, antimicrobial agents, antioxidants, and engineering property enhancers (Mohamad et al., 2022; Rahmiati et al., 2024), most studies remain limited to one or two plant-derived additives and a narrow range of field parameters. Therefore, comprehensive data regarding the effects of various natural additives on microbial stability, antioxidant capacity, physicochemical properties, and processing parameters in watermelon juice are scarce. Furthermore, the integration of artificial neural network (ANN) modeling with traditional statistical methods, to evaluate multiple quality parameters of naturally preserved fruit juice has been inadequately investigated. This research’s central contribution is the concurrent investigation of the bioactive compounds present in clove, cinnamon, bay leaf, and ginger rhizomes, in combination with lime juice and honey, as single and multi-component preservative systems for watermelon juice, while concurrently analyzing microbial, antioxidant, phytochemical, physicochemical, thermo-rheological, and electrical properties. The ANN modeling was integrated as an auxiliary predictive method, for investigating nonlinear correlations between the input features, and the targeted engineering and microbial outcomes. The developed models serve as a proof-of-concept foundation for data-driven forecasting under the tested experimental parameters.

## Materials and methods

### Reagents and apparatus

Otherwise stated, all the solvents used to achieve the experimental framework, were analytical-grade and manufactured by Merck (Darmstadt, Germany). Sodium benzoate (C_7_H_5_NaO_2_), with a molar mass of 144.11 g/mol and 99% purity, was produced by Thermo Fisher Scientific Inc., based in America. Also the plate count agar (PCA), and potato dextrose agar (PDA), were manufactured by Thermo Fisher Scientific Inc., based in America. Analytical grade powder of sucrose (≥99.5%), gallic acid, quercetin, ascorbic acid, DPPH, lycopene (≥98%) and β-carotene (≥95%) were purchased from Sigma-Aldrich (St. Louis, MO, USA).

### Watermelon sampling and juice preparation

Matured watermelons (*Citrullus lanatus*) were obtained from a local farm in Saudi Arabia. The fruits were manually inspected, to remove rotten and pest-infected fruits, then thoroughly washed with tap water and dried with sterile gauze. These cleaned fruits were manually rinded under aseptic conditions, dice, blended with a laboratory blender, and sieved through a 150 μm mesh sieve to obtain watermelon juice. The fresh juice obtained was poured into amber-colored glass bottles, wrapped with aluminium foil, and kept in a cool dark environment, prior to further treatment.

### Plant extracts preparation

The extracts were prepared through the maceration extraction process. The plant materials were dried and ground using a laboratory milling machine, and sieved through a sieve with a number 200 mesh. Then, 500 g of the sieved material was added to 2 L of food-grade ethanol (97% purity) and kept at room temperature (20–31 °C) for four days, with the mixture stirred intermittently for 30 min every 8 h. After four days, the mixture was filtered with a Whatman filter paper, and the filtrate was concentrated by evaporation by using the water bath at 45 ± 2 °C, until the ethanol was evaporated (Bitwell et al., 2023). The extract recovered was stored in sterile glass bottles, and kept at 15 °C until further notice use.

### Samples preparation and basis of experimental design framework

The experimental mix design ratio used for this study is presented in Table 1. The natural additives used in this research were not selected randomly, but rather based on their diverse and wide range of phytochemicals concentrations, acidity levels, natural sugars content, and engineering properties. Clove (*Syzygium aromaticum*) seeds contain a large amount of eugenol, vitamin C, terpenoids, and flavonoids, resulting in its potent antimicrobial and antioxidant properties (Abdelmuhsin et al., 2025). Cinnamon (*Cinnamomum verum*) bark is rich in cinnamaldehyde and benzaldehyde (Yanakiev, 2020). Bay (*Laurus nobilis*) leaf contains large amounts of phenolics and terpenoids, which have strong antioxidant, antimicrobial, and neuroprotective properties (Awada et al., 2023). Ginger (*Zingiber officinale*) rhizome is packed with appreciable amounts of terpenoid compounds, gingerols, and shogaols, which possess documented antioxidant, antimicrobial, and preservative capabilities (Bischoff-Kont and Fürst, 2021). The lime juice contains appreciable concentrations of ascorbic acids, flavonoids, and saponins, which are potent antioxidants and antimicrobial compounds. Also, the high acidity and elevated electrical conductivity level of lime juice, helps to increase the acidity and ionic composition of the treated juice specimens, thus impairing microbial metabolism (Oikeh et al., 2015). Furthermore, honey contains significant natural sugars, as well as fair amounts of organic acids, proteins, and minerals. These sugars have the ability to reduce the water activity of the juice, leading to disruption of the microbial cell membrane, and leaking of the intracellular components – hence inhibiting food spoilage. Similarly, the sugar content affects the juice’s viscosity and thermal behavior, ultimately leading to the rheological modification (Pląder et al., 2025).

**Table 1:** Experimental mix design ratio.

Treatment code	Sodium benzoate (0.25 g; final conc.–0.1% w/v)	CSE (mL)	CiE (mL)	BLE (mL)	GRE (mL)	Lime (mL)	Honey (mL)	WJ (mL)
NC	0	0	0	0	0	0	0	250
PC*	0.25 g (0.1% w/v)	0	0	0	0	0	0	250
T1	0	5	0	0	0	0	0	245
T2	0	0	5	0	0	0	0	245
T3	0	0	0	5	0	0	0	245
T4	0	0	0	0	5	0	0	245
T5	0	0	0	0	0	5	0	245
T6	0	0	0	0	0	0	5	245
T7	0	2.5	2.5	0	0	0	0	245
T8	0	0	2.5	0	0	0	2.5	245
T9	0	2.5	0	0	0	2.5	0	245
T10	0	0	2.5	2.5	0	0	0	245
T11	0	1.5	1.25	1.5	1.25	0	2.0	242.5
T12	0	1.25	2.5	2.5	1.25	0	0	242.5
T13	0	2.5	2.5	2.5	1.25	7	2.5	231.75
T14	0	1.25	1.75	1.25	1.5	0	2.5	241.75
T15	0	1.25	1.25	2.5	2.5	7	0	235.5
T16	0	1.75	2.5	2.5	2.5	0	0	240.75

*, The PC was produced upon solubilizing 0.25 g sodium benzoate directly into the measured watermelon juice; CSE, clove seed extract, CiE, cinnamon extract, BLE, bay leaf extract, GRE, ginger rhizome extract, WJ, Watermelon juice, PC, positive control, NC, Negative control, T1 to T16, treatments 1 to 16.

Sodium benzoate (C_7_H_5_NaO_2_) is a common artificial food preservative globally, known for causing fewer health complications when applied within established dosage limits. 0.25 g of C_7_H_5_NaO_2_ was dissolved in 250 mL of the watermelon juice, and was used as the positive control, to produce C_7_H_5_NaO_2_ additive concentration of 0.1% (w/v). This preservative concentration, was chosen based on prior research findings (Mehmood et al., 2008; Alam et al., 2013; FDA, 2026). All the solutions used this study were prepared using distilled water.

The experimental mix design ratio was designed to answer the research question, which is whether natural preservatives scheme can enhance the antimicrobial, antioxidant, and engineering properties of watermelon juice, as well as provide dependable datasets for predictive ANN modeling. Eighteen experimental units (including negative and positive controls) were set up within a completely randomized experimental design. Particularly, treatments 1 to 6 were one-way (component) units, while treatments 7 to 16 were either two-component or multi-component systems. All the specimens were formulated based on the mix design ratio presented in Table 1, and they were poured aseptically into sterilized containers, sealed properly, and stored under laboratory conditions as explained in the next subsection.

### Samples storage condition

Temperature, oxygen, and UV light are the major environmental factors, that affect fruit juices (including watermelon juice) stability during storage (Bhattacharjee et al., 2019). Therefore, based on previous studies’ recommendations (Liu et al., 2018; Mohamad et al., 2022), the watermelon juice samples used in this study were kept at a controlled storage condition (20 ± 2 °C), in a dark cabinet (protected from direct UV light). This is to maintain a stable, controlled temperature environment for assessing the temporal impact of treatments on microbial activity, thermo-rheological behavior, and electrical characteristics. The microbial, thermo-rheological and electrochemical properties, of all the specimens were analyzed on storage day 0, 2, 4, 6, 8, and 10. The experimental observation period of 10 days, use in this research was designed for controlled study purposes, distinct from determining the product’s market shelf life. Furthermore, the sealed bottles were allocated to each sampling time point to prevent multiple openings and sampling of the identical container, thereby accurately representing the experimental protocol. Three independent bottles were used as biological replicates for each treatment and storage interval. Particularly, this study did not include structured sensory assessments with human panelists.

## Laboratory analysis

### Total phenolic content (TPC) evaluation

The TPC was measured through the procedure explained by Singleton and Rossi (1965), by using the Folin–Ciocalteu spectrophotometric technique. 0.3 mL of the sample was added to 1 mL of distilled water and 1 mL of Folin–Ciocalteu reagent (10% v/v), and kept at room temperature for 10 min. Then, 1 mL of Na_2_CO_3_ solution (7.5% w/v) was mixed with the sample and kept at 25 ± 2 °C for 60 min. Then, 0.5 mL of the product, blank samples, and standard (Gallic acid) samples were pipetted into a 96-well plate, incubated at 25 ± 2 °C for 30 min, and their absorbance was measured at 765 nm using a UV–Visible spectrophotometer. The results were expressed as gallic acid equivalents (GAE) per 100 mL of the sample (extract or juice).

### Total flavonoid content (TFC) determination

The TFC was determined through the Aluminum chloride (AlCl₃) colorimetric approach, by using the procedure described by Zhishen et al. (1999). 1 mL of the sample was incorporated into a mixture consisting of 5 mL of CH_3_OH, 0.3 mL of AlCl_3_ (10% w/v), 0.3 mL of 1 M C_2_H_3_KO_2_, and 10 mL of distilled water. The product was extensively agitated, and the absorbance was read at 490 nm, with the aid of a UV–Visible spectrophotometer. Then, the TFC value of each sample was determined by using a calibration curve prepared from quercetin reference solutions (da Silva et al., 2015). The results were expressed as mg quercetin equivalents (QE) per 100 mL of the sample (extract or juice).

### Ferric reducing antioxidant power (FRAP) determination

The FRAP level in the samples was measured, in accordance with the technique explained by Benzie and Strain (1996), using Trolox as the standard reference material. The FRAP solution was formulated by combining 0.3 M acetate buffer (pH 3.6), 10 mM TPTZ reagent prepared in 40 mM HCl, and 20 mM FeCl_3_ solution in a ratio of 10:1:1, respectively. Subsequently, 0.2 mL of the samples was mixed with 2 mL of the FRAP solution, and kept at 37 ± 1 °C for 60 min. The absorbance was then read with the aid of a UV–Visible spectrophotometer at 593 nm. Ferrous sulfate served as the standard reagent for constructing the calibration curve, and the results were expressed as μmol Fe^2+^/mL.

### DPPH radical scavenging assay determination

The DPPH assay of the samples was determined by a slightly modified method explained by Brand-Williams et al. (1995). 4 mL of freshly prepared DPPH solution (0.06 mM) was added to 1 mL of the sample, mixed vigorously, and held at 25 ± 2 °C for 40 min. Then, a UV–Visible spectrophotometer was used to measure the absorbance at 517 nm.

### Total soluble solids and pH

The pH of the fruit juice was measured in accordance with AOAC (2023) Official Method of Acidified Foods. The digital pH meter (model no: PH-3C) manufactured by WANT Balance Instrument Co., Ltd., China, was calibrated with buffer solutions (pH 4.0 and 7.0) before it was inserted into the juice sample, and the pH value was taken after a stable reading.

The total soluble solids (TSS) level in the samples was measured with a digital refractometer (model: RX-7000α, produced by ATAGO Co., Ltd.), in accordance with AOAC Official Method 983.17 (2023). The instrument was calibrated with distilled water, and the TSS readings were reported as °Brix.

### Vitamin C determination

The Vitamin C concentration in the samples was measured through the UV–Visible spectrophotometric approach, by adopting the AOAC standard guidelines. The specimen was sieved using a fine membrane filter, and quantification was done using certified reference material (ascorbic acid), within a concentration range of 0 to 50 mg/L. The absorbance of the prepared sample was measured with a UV–Visible spectrophotometer at 265 nm. Linear regression analysis was conducted between concentration and absorbance, and the coefficient of determination (*R*^2^) obtained was greater than 0.990, suggesting outstanding linearity (Andaririt et al., 2025). Then the vitamin C was computed as mg/100 mL.

### Electrical conductivity and electrical resistivity

The electrical conductivity and electrical resistivity of the samples were determined according to ASTM D1125 (2023) protocols. The conductivity was measured directly (probe immersion) using a conductivity meter (Model DDS -11A, produced by HENWI Technology, China); while the resistivity was calculated using Equation 1. Before the test, the machine probe was calibrated with potassium chloride solution, the probe was cleaned with distilled water, and the measurements were taken at 26 ± 3°. The sample was devoid of air bubbles, when the electrical conductivity (EC) readings were taken.


$$\rho =\frac{1}{\sigma }$$ (1)

Where: ρ is the electrical resistivity, and σ is the electrical conductivity. The electrical conductivity (EC) was expressed as mS/cm, and the electrical resistivity was expressed as Ωm.

### Viscosity and density

The specimen rheological characterization was determined according to ASTM D2196 (2020) procedures. The juice flow behavior (viscosity) was measured using a HAAKE falling ball viscometer, produced by Thermo Fisher Scientific Inc. in America, with a viscosity range of 0.5 to 1,000,000 cSt and a temperature range of −20 °C to +150 °C.

The density was measured in accordance with ASTM D1480 (2021) guidelines using a standard pycnometer bottle. A dry bottle with a measured mass and volume was packed with the juice, and the new mass was recorded before calculating the density using Equation 2.


$$\text{Density}=\frac{m}{V}$$ (2)

Where m is the mass of the juice sample, and V is the pycnometer volume.

Both tests were conducted under laboratory temperature of 26 ± 3 °C. The viscosity was expressed in mm^2^/s; while the density was expressed in kg/m^3^.

### Thermal conductivity and specific heat capacity

The thermophysical characterization (thermal conductivity) of the samples was determined in accordance with the ASTM D7896 (2019) approved procedure, in order to estimate the heat flow dynamics as affected by the treatments. A digital thermal conductivity tester (model CY-809S, accuracy ±3%, repeatability error ≤3%), produced by Jinan Cyeeyo Instruments Co., Ltd., in Shandong Province, China, was used for the thermal conductivity testing. At each test, 100 mL of the specimen was placed into a chamber, the machine probe was inserted into the liquid, and the readings were taken from the machine screen, after reaching thermal equilibrium. The test was conducted at laboratory temperature, and the probe was washed with distilled water and dried with hot air after each test. The thermal conductivity (λ) was expressed in W/m. K.

Furthermore, the specific heat capacity (Cp) was determined in agreement with ASTM E1269 (2018) guidelines. An automatic high-temperature differential scanning calorimeter (DSC), with a precision of ±0.02 K, produced by Thermo Fisher Scientific Inc., America, was used to carry out the specific heat capacity evaluation. About 10 mg of the specimen were sealed airtight into an aluminum pan, placed into the DSC machine, and heated at a regulated temperature, and nitrogen atmospheric condition. The reference was a plain aluminum pan, and the electronic component of the machine computes the Cp values, which are then expressed in kJ/kg·K.

### Shear stress and dielectric constant (ɛ)

The samples shear stress was measured through ASTM D2196 (2020) standard, using a Thermo Fisher HAAKE™ MARS™ iQ Rotational Rheometer, manufactured by Thermo Fisher Scientific, America. 200 mL of the sample was poured into the rheometer container, and the shear rate was achieved by methodically adjusting the spindle velocity. The equivalent shear stress was then calculated.

The dielectric characterization (dielectric constant) of the juice samples was determined using the ASTM D150 (2018) procedures and an LCR meter (model: TECPEL LCR-2100) manufactured by TECPEL Co., Ltd., Taiwan. Specifically, the test was conducted at 10 kHz and 26 ± 2 °C, with the samples free from air bubbles.

## omparative integrity and efficacy indices

### Nutritional and phytochemical bioavailability index (NPBI)

The NPBI of the extracts and treatments was calculated using the nutritional/phytochemical synergy index approach, as shown in Equation 3.


$$\text{NPBI}=\frac{\frac{x_{1}}{x_{1}\mathrm{NC}}+\ldots +\frac{x_{n}}{X_{\mathrm{nNC}}}}{N}$$ (3)

Where: *N*–total number of parameters investigated, x_1_ to x_n_–the individual parameter investigated, NC- the value recorded for the NC unit (Santana Andrade et al., 2022).

In this research, NPBI was considered as a relative measure of nutritional and phytochemical efficacy. Values exceeding 1 denote a relative enhancement compared to the reference condition, whereas a value of 1 signifies equivalent performance, and values below 1 indicate inferior performance. These metrics served to evaluate the general nutritional and phytochemical effects of the interventions and should not be regarded as conclusive proof of synergism among the additives.

### Modified antioxidant capacity index (MACI)

The MACI values, which reflect the antioxidant performance, were calculated with a modified model shown in Equation 4.


$$\text{MACI}=\frac{T_{\text{treatmnet}}}{T_{\text{Expected}}}$$ (4)

The computed MACI values served to compare the efficacy of the formulations rather than to confirm true biochemical synergy. Therefore, elevated MACI scores were considered indicative of enhanced antioxidant capacity within the experimental parameters, while the metrics alone did not provide enough evidence for interactions–be they synergistic, additive, or antagonistic–between components (Putra et al., 2021).

### Fractional inhibitory concentration index (FICI)

Since the minimum inhibitory concentration (MIC) was not established in this study, the FICI was modified to microbial inhibition efficiency (MIE). Consequently, the calculated synergy index (SI) and MIE derived through Equations 5, 6, were interpreted as a modified comparative preservation index rather than a conventional FICI-based synergy metric.


$$\mathrm{MIE}=\frac{\mathrm{TM}C_{\mathrm{NC}}-\mathrm{TM}C_{\text{sample}}}{\mathrm{TM}C_{\mathrm{NC}}}\times 100$$ (5)

$$\mathrm{SI}=\frac{\mathrm{MI}E_{\text{sample}}}{\text{best sample}\;\mathrm{MIE}}$$ (6)

Values of SI close to or greater than 1 were interpreted as signs of enhanced preservation capacity, within this computational model; nonetheless, they did not constitute conclusive evidence of synergistic or antagonistic antimicrobial activity (Ormeneanu et al., 2025). Notably, the NPBI, MACI, MIE and SI indices served as benchmarking metrics to assess the relative preservation efficacy of the tested additive formulations. These indices allowed for the comparison of treatments based on microbial reduction and preservation outcomes under experimental storage conditions. Rather than acting as definitive pharmacological or microbiological indicators of synergism, their main goal was to establish a uniform basis for evaluating treatment efficacy in experimental settings (Natarajan et al., 2019).

## Microbial determination

### Total bacterial count (TBC) and total fungal count (ToFC) evaluation

At each specified sampling point, a sterile aliquot was collected from each container for microbiological assessment, resulting in *n* = 3 independent biological replicates per treatment condition and storage duration The TBC was determined in accordance with the ISO 4833-1 (2013) approved procedure, while the ToFC was determined in agreement with ISO 21527-1 (2008) guidelines. The juice sample (1 mL) was aseptically added to 9 mL of sterile isotonic saline (0.85% NaCl) to produce a 10^−1^ dilution, followed by serial 10-fold dilutions. This is to achieve appropriate dilution levels for the TBC and ToFC loads. All microbiological evaluations were conducted under sterile conditions.

To measure the TBC level in the dilution, 0.1 mL aliquot of the 10^−1^ to 10^−5^ dilutions was spread on a PCA plate and kept at 30 ± 1 °C for 2 days, after which the formed clusters were counted with the aid of a digital colony counter. The results were expressed as CFU/mL. To measure the ToFC level, 0.1 mL of the portion was spread on chloramphenicol treated PDA plate, and incubated at 27 ± 1 °C for 4 days, after which the fungal clusters produced were counted and expressed as CFU/mL. The quantifiable range for fungi was 10–130 CFU per plate, whereas the quantifiable range for TBC was 20–200 CFU per plate. Additionally, the detection limit for both the TBC and ToFC were 100 CFU/mL.

### Artificial neural network development

The artificial neural network (ANN) model was conducted with the MATLAB software (MathWorks, Natick, MA, USA). For this study, a multilayer perceptron ANN was created to forecast the selected response variables – electrical conductivity, viscosity, total bacteria count, and total fungal count, which were selected based on their wider application in the public health and engineering sectors. The system comprises of train-validation-test split. The treatments were coded NC, PC, T1 to T16,

The dataset contained 12 features: storage period, treatment, electrical conductivity (EC), electrical resistivity (ρ), viscosity, density, thermal conductivity, specific heat capacity, shear stress, dielectric constant, total bacterial count (TBC), and total fungal count (ToFC). For each Artificial Neural Network model, one of these variables was assigned as the prediction target (label), while the other 11 variables served as input features (predictors or explanatory variables). Consequently, the input layer of the ANN consisted of 11 neurons (nodes), with the particular output neuron (target node) varying depending on the specific property (response variable or dependent variable) being modeled or forecasted. Notably, since ρ was computed directly from EC (Equation 1), including it as a feature during EC prediction would introduce data leakage. Consequently, the ρ parameter was excluded from the EC prediction model, leaving 10 input variables for EC projection.

All networks were trained and optimized using the Levenberg–Marquardt algorithm (trainlm). The model parameters (weights and biases) were optimized throughout the training process, to reduce the error between observed and predicted outputs. The predefined data scaling process in MATLAB was employed to normalize input and target data into an appropriate numerical scale before network training. The dataset was partitioned into 70% training, 15% validation, and 15% test subsets. The validation subset was utilized during training to assess model generalization and to determine early stopping criteria, while the test subset was reserved for final performance evaluation. The training set was employed to optimize the network parameters, while the validation set was utilized during training to assess model generalization and identify the early termination point. The evaluation subset was preserved for unbiased evaluation of model accuracy on the partitioned dataset. Plots of projected versus actual values and error histograms were analyzed to evaluate the model’s performance, precision, and error distribution. The *R*^2^ was derived from the correlation between the predicted outputs and the laboratory results. Then, the selected neural network models were chosen based on their accuracy and training stability. Linear regression and ANN modeling served complementary roles: linear regression offered an interpretable analysis of the relationship, between treatment or storage duration and the observed responses; while ANN modeling was employed to capture potential nonlinear dependencies between the input features and the output variables, instead of being the principal originality of the research. Model efficacy was evaluated using the coefficient of determination (*R*^2^) and root mean squared error (RMSE).

### Statistical analysis

This study’s results were evaluated using IBM SPSS Statistics 20.0 software. The effect of the treatments on watermelon juice parameters and stability was evaluated using Analysis of Variance (ANOVA). For each treatment and storage duration, three separate bottles served as biological replicates (*n* = 3). Analysis was conducted via the General Linear Model–Multivariate approach in SPSS, treating treatment and storage period as fixed factors and modeling their interaction. Duncan’s Multiple Range Test was employed to identify statistically significant differences among means at the 5% significance threshold. Additionally, linear regression analyses were used, to evaluate the relationships between the treatment or storage period, and antioxidant parameters, phytochemicals, and microbial actions; subsequently, ANN was employed as an additional nonlinear forecasting technique. The experiments were performed in triplicate, and the results are expressed as the mean ± standard deviation. All the tests were done at laboratory temperature of 25 ± 3 °C.

## Results and discussion

### The antioxidant assay, phytochemicals, physicochemical characteristics, and engineering properties of the additives

Table 2 presents the results of the antioxidant assays, physicochemical attributes, phytochemicals, and engineering properties of the additives used in this experimental framework, for the watermelon preservation. Among the natural additives, it was noted that the plants’ extracts (CSE, CiE, GRE, and BLE) exhibited the maximum antioxidant potential, which may be linked to the abundance of phytochemicals present in these extracts. According to previous reports, clove seeds, cinnamon, bay leaves, and ginger rhizomes are rich sources of phytochemicals, including cinnamaldehyde, cineole, gingerols, shogaols, eugenol, gallic acid, and tannins, which are potent antioxidant compounds (Cortés-Rojas et al., 2014; Mao et al., 2019; Solanki et al., 2025). These phytochemicals have the potential for free radical scavenging activity; hence, they may inhibit oxidative degradation of biomaterials, including fruit juices (Hamdoun et al., 2026).

**Table 2:** The antioxidant assay, phytochemicals content, physicochemical characteristics, and engineering properties of the additives.

Parameter	Additives						
	C_7_H_5_O_2_Na (0.1% w/v)	CSE	CiE	BLE	GRE	Lime juice	Honey
DPPH (%)	5.32^a^ ± 0.16	91.00^f^ ± 2.2	92.57^f^ ± 0.38	89.92^d^ ± 1.15	93.56^e^ ± 0.39	75.72^c^ ± 0.55	65.12^b^ ± 0.33
TPC (mg GAE/100 mL)	0.00^a^ ± 0.00	510.61^g^ ± 1.21	460.06^f^ ± 1.01	354.27^d^ ± 0.87	400.56^e^ ± 1.17	116.7^9c^ ± 1.48	91.03^b^ ± 1.68
TFC (mg QE/100 mL)	0.00^a^ ± 0.00	162.99^g^ ± 0.81	138.13^f^ ± 1.88	113.56^d^ ± 2.89	125.91^e^ ± 2.33	27.13^c^ ± 0.25	17.04^b^ ± 0.67
FRAP (μmol Fe^2+^/mL)	0.13^a^ ± 0.03	26.63^g^ ± 0.38	23.89^f^ ± 1.07	18.52^d^ ± 0.33	19.93^e^ ± 1.51	8.64^c^ ± 0.20	6.15^b^ ± 0.13
VC (mg/100 mL)	0.00^a^ ± 0.00	1.79^c^ ± 0.02	1.03^b^ ± 0.05	1.42^bc^ ± 0.05	6.58^d^ ± 0.18	52.00^e^ ± 0.70	1.25^b^ ± 0.04
pH	7.69^f^ ± 0.20	5.65^cd^ ± 0.12	5.48^c^ ± 0.08	6.17e ± 0.06	5.74^d^ ± 0.09	2.46^a^ ± 0.04	4.21^b^ ± 0.02
TSS (°Brix)	0.09^a^ ± 0.02	3.07^b^ ± 0.08	2.81^b^ ± 0.03	2.42^b^ ± 0.05	2.92^b^ ± 0.04	9.35^c^ ± 0.12	88.09^d^ ± 1.19
EC (mS/cm)	1.08^a^ ± 0.02	3.65^e^ ± 0.09	3.33^d^ ± 0.03	3.03^c^ ± 0.06	3.44^d^ ± 0.07	5.76^f^ ± 0.10	2.60^b^ ± 0.05
λ (W/m·K)	0.63^d^ ± 0.03	0.51b ± 0.03	0.54^bc^ ± 0.01	0.56^c^ ± 0.02	0.52^b^ ± 0.02	0.64^d^ ± 0.02	0.41^a^ ± 0.02
Viscosity (mPa·s)	0.98^a^ ± 0.03	2.28^e^ ± 0.07	2.14^d^ ± 0.03	1.92^c^ ± 0.12	2.08^d^ ± 0.03	1.16^b^ ± 0.02	9.45^d^ ± 0.10
ɛ	70.61^d^ ± 0.61	47.61^b^ ± 0.47	45.24^b^ ± 0.4	49.72^b^ ± 0.70	48.50^b^ ± 0.20	65.49^c^ ± 0.45	38.89^a^ ± 0.30

Mean ± standard deviation; *n* = 3; λ–thermal conductivity; ɛ–dielectric constant; EC–electrical conductivity; VC–vitamin C; TSS–total soluble solids; CSE–clove seed extract; CiE–cinnamon extract; BLE–bay leaf extract; GRE–ginger rhizome extract; Means with similar letter within the same row do not differ significantly (*p* ≤ 0.05), according to DMRT.

Also, the lime juice exhibited the maximum vitamin C concentration, coupled with the highest acidity content (lowest pH value). Apart from the antimicrobial effect of the high vitamin C and acidity levels, vitamin C is a good corrosion inhibitor (Irwan and Suhendrayatna, 2019); hence, it may prevent the corrosion of the metallic parts of fruit juice processing machines. Likewise, the lower pH level of the lime juice may enhance the electrical conductivity of the preserved juice samples and potentially improve biosensor and control systems design. Assawarachan and Tantikul (2025) stated that there is a strong positive correlation, between acidity and higher electrical conductivity, and this relationship is helpful in developing sustainable models for food processing and preservation. Additionally, honey has the highest TSS and viscosity values, and this may have advantageous engineering implications, beyond nutritional and medical applications. Blending watermelon juice with honey may increase the total soluble solids, and viscosity of the enriched juice sample. These two parameters (TSS and viscosity) provide crucial data for the modeling and design of fruit juice processing system pipelines, pumping machines, and dielectric sensing (Tian et al., 2007; Xiong et al., 2025).

The TPC and FRAP values obtained in the study for the natural additives were lower than the results documented by Heckmann et al. (2024) for screened plant extracts. The vitamin C content recorded for honey in this study was less than the values documented by Zawawi et al. (2021), although the TPC and FRAP values aligned with their results for honey samples. Moreover, the TPC and TFC values obtained in this research were less than the results, obtained by Liew et al. (2018) for *C. sinensis* peel extracts. Notably, this study’s TPC, TFC, and vitamin C results were generally greater than those reported by Abeysuriya et al. (2020) for several fruit juices. The discrepancies observed between this study’s results and previous authors’ findings, can be attributed to differences in bee species, plant genotypes, soil conditions, environmental pollution, plant materials, maturity stages, and analytical techniques employed by different researchers.

### Physicochemical characteristics, antioxidant assay, and antioxidant phytochemicals of the blended juice samples

Table 3 presents the results of the impact of the natural derivatives on the watermelon juice samples’ physicochemical properties, antioxidant activity, and phytochemicals. The findings demonstrate that the synthetic and natural derivatives have a significant impact on the watermelon juice’s physicochemical quality, antioxidant assay, and phytochemicals content. The substantial enhancement observed in the antioxidant activity and physicochemical properties of the juice after treatment can be attributed to the quality (measured by DPPH, TPC, TFC, and FRAP assays) of the natural additives used (Table 3). Based on their mix-design categories (one additive, two-additive combination, and multi-additive combination), T1 showed the maximum antioxidant enhancement in the individual extract category, while T13 demonstrated the highest antioxidant performance in the multi-additive combination. The observed better preservative effect of T3 may be linked to the combined consequence of formulation composition, additive dose, acidification, and the physicochemical properties of the individual components. Notably, the lime juice caused an increase in Vitamin C, and acidity levels of the watermelon juice, as seen in T5, T9, T13, and T15 samples. This may be linked to the elevated ascorbic acid content, and lower pH of the lime juice (Table 3). According to Rahmiati et al. (2024), lime juice contains a remarkable amount of ascorbic acid and organic acids, which may enhance food antioxidant quality and acidity.

**Table 3:** The physiochemical characteristics and antioxidant phytochemicals of the juice samples.

TC	DPPH (%)	TPC (mg GAE/100 mL Juice)	TFC (mg QE/100 mL Juice)	FRAP (μmol Fe^2+^/mL)	Vitamin C (mg/100 mL)	pH	TSS (°Brix)
NC	37.87^a^ ± 1.06	48.09^a^ ± 0.23	19.54^a^ ± 0.34	3.00^a^ ± 0.05	9.14^b^ ± 0.05	5.63^k^ ± 0.15	9.95^f^ ± 0.04
PC	39.95^a^ ± 0.19	51.65^b^ ± 0.57	20.23^b^ ± 0.48	3.17^b^ ± 0.05	8.63^ab^ ± 0.19	5.45^j^ ± 0.03	9.23^c^ ± 0.03
T1	63.26^ef^ ± 0.37	110.70^h^ ± 0.30	44.71^g^ ± 0.39	6.48^g^ ± 0.12	9.34^b^ ± 0.08	5.34^hi^ ± 0.04	9.15^c^ ± 0.04
T2	59.15^de^ ± 0.07	101.51^g^ ± 0.46	41.80^d^ ± 0.33	5.82^f^ ± 0.12	8.15a ± 1.77	5.26^gh^ ± 0.04	9.10^bc^ ± 0.02
T3	54.30^bc^ ± 0.07	91.77^e^ ± 0.18	37.92^e^ ± 0.16	5.44^e^ ± 0.12	9.04^b^ ± 0.03	5.42^ij^ ± 0.04	9.20^c^ ± 0.02
T4	57.48^cd^ ± 0.15	98.63^f^ ± 0.41	40.85^d^ ± 0.91	5.74^f^ ± 0.03	9.20^b^ ± 0.06	5.34^hi^ ± 0.02	9.12^bc^ ± 0.02
T5	50.32^b^ ± 0.12	84.82^c^ ± 0.16	30.58^c^ ± 0.58	4.89^c^ ± 0.12	15.30^g^ ± 0.46	4.07^a^ ± 0.05	8.87^b^ ± 0.05
T6	53.13^bc^ ± 0.11	89.96^d^ ± 0.57	33.44^d^ ± 0.35	5.16^d^ ± 0.07	11.05^cde^ ± 0.11	5.13^de^ ± 0.03	10.26^g^ ± 0.05
T7	70.75^h^ ± 0.22	128.84^l^ ± 0.60	51.48^i^ ± 0.48	7.50^j^ ± 0.05	9.46^b^ ± 0.08	5.27^gh^ ± 0.06	9.16^c^ ± 0.06
T8	67.47^fgh^ ± 0.48	119.65^j^ ± 0.77	47.82^h^ ± 0.56	6.92^h^ ± 0.08	10.69^cd^ ± 0.24	5.16^ef^ ± 0.02	9.64^e^ ± 0.03
T9	64.96^fg^ ± 0.14	113.02^i^ ± 0.51	42.42^fg^ ± 0.47	6.58^g^ ± 0.07	14.91 g ± 0.11	4.91^b^ ± 0.03	9.25^cd^ ± 0.57
T10	68.47^gh^ ± 0.53	124.00^k^ ± 0.41	49.19^hi^ ± 0.16	7.15^i^ ± 0.07	9.33^b^ ± 0.12	5.24^fg^ ± 0.03	9.13^bc^ ± 0.03
T11	77.88^ij^ ± 0.17	151.50^n^ ± 0.47	62.21^k^ ± 0.33	8.59^l^ ± 0.15	11.43^de^ ± 0.28	5.06^cd^ ± 0.05	9.56^e^ ± 0.05
T12	75.83^i^ ± 0.25	144.75^m^ ± 0.45	57.74^j^ ± 0.28	8.17^k^ ± 0.05	10.60^c^ ± 0.21	5.23^fg^ ± 0.04	9.09^bc^ ± 0.03
T13	91.30^l^ ± 1.62	191.86^r^ ± 0.23	77.31^n^ ± 0.23	10.37^p^ ± 0.1	17.82^h^ ± 0.13	4.02^a^ ± 0.05	8.63^a^ ± 0.05
T14	79.80^ijk^ ± 0.25	158.12^o^ ± 0.19	64.02^kl^ ± 0.23	8.80^m^ ± 0.14	12.25^f^ ± 0.07	5.02^c^ ± 0.06	9.48^de^ ± 0.04
T15	86.15^kl^ ± 0.46	178.54^q^ ± 0.50	72.39^m^ ± 0.27	9.72^o^ ± 0.05	17.09^h^ ± 0.11	4.08^a^ ± 0.03	8.61^a^ ± 0.05
T16	81.58^jk^ ± 0.58	162.67^p^ ± 0.72	64.97^l^ ± 2.86	9.09^n^ ± 0.03	11.75^ef^ ± 0.07	5.14^de^ ± 0.03	9.04^bc^ ± 0.04

Mean ± standard deviation; *n* = 3; Means with similar letter within the same column do not differ significantly (*p* ≤ 0.05), according to DMRT; TC–treatment code.

The boost in the antioxidant assay and phytochemicals, observed in the treated watermelon juice, mainly the multi-component combination, may represent the cumulative activity of bioactive constituents in diverse natural adjuncts, use for the juice fortification. Previous reports have stated that bioactive compounds–such as phenolics, flavonoids, terpenoids, gingerols, cinnamaldehyde, and eugenol, obtained from clove, cinnamon, bay leaf, ginger, lime, and honey, can provide a broad spectrum of antioxidants and phytochemicals (Mao et al., 2019; Diniz do Nascimento et al., 2020). Additionally, the higher TSS results recorded for the honey-based treatments could be linked to the higher concentrations, of glucose and fructose in natural honey. These compounds have the potential to increase the soluble solids in fruit juices (Sadeghi et al., 2019).

Generally, elevated antioxidant activity and phytochemicals, recorded in this study may lead to possible enhanced shelf-life, consumer acceptability, and engineering properties of the treated juice. According to previous reports (Losada-Barreiro et al., 2022; Yang and Ling, 2025), antioxidants and phytochemical compounds combine their bioactive properties, to improve the anti-oxidation and anti-degradation of food systems’ biochemical and engineering properties. Interestingly, the elevated levels of DPPH, TPC, TFC, vitamin C, and FRAP–documented in the T11, T13, and T15 samples, may be linked to cooperative effects of numerous bioactive compounds, enhanced phytochemicals, carboxylic acids derived from lime juice, honey osmotic influence, and potential cooperative effects among the Organic supplements. This may result in greater free radical scavenging ability and anti-microbial activity, thereby impairing oxidative reactions and microbial spoilage. It also helps preserve the juice’s essential properties, such as rheological behavior and other critical electrical parameters, which is one of the major goals of this experimental framework. Based on the results, the pH and TSS data can offer great insight, into the development of juice production and quality control systems (Basak et al., 2022; Polak et al., 2024), while the other parameters (DPPH, TPC, TFC, vit. C, and FRAP) can be used in the design of food preservation systems.

### Thermo-rheological and electrical properties behavior of the watermen juice during storage

Tables 4, 5, and Supplementary Table S1 present that effect of treatment and storage duration on the engineering properties of the watermelon juice. The multivariate ANOVA revealed that, both storage duration and treatment type have a significant influence on the investigated engineering parameters (*p* ≤ 0.05). Generally, there were alterations in the engineering parameters during storage, which was more pronounced in the untreated juice sample, compared to the treated juice samples. This may be attributed to the elevated antioxidant levels in the treated juice (Table 3), which have the potential to inhibit oxidative degradation of the engineering properties, and to enhance the physicochemical stability of the juice as storage progresses. Previous reports have reported that antioxidant phytochemicals (such as those stated in Tables 2, 3), tend to impair redox reactions that contribute to quality deterioration, and microbial propagation (Gulcin, 2025; Tüğen and Buruleanu, 2025). This may suppress extracellular metabolite production and biofilm formation, which can compromise juice stability during storage. The higher EC values recorded in the treatments containing lime juice, may be attributed to the lime juice higher citric acid and minerals’ content (Das and Sahu, 2022), which has the potential of increasing the ion mobility and conductive electrolyte of the blended watermelon juice.

**Table 4:** The engineering properties of the watermelon juice at Day 0.

TC	EC (mS/cm)	Resistivity (Ω·m)	Viscosity (mPa·s)	Density (kg/m^3^)	λ (W/m·K)	Cp (kJ/kg·K)	Ʈ (Pa)	Dielectric constant
NC	3.91^a^ ± 0.04	2.56^m^ ± 0.03	1.95^a^ ± 0.04	942.56^a^ ± 11.30	6.87 ×10^-1k^ ± 0.01	4.62^l^ ± 0.04	0.59^a^ ± 0.02	85.56^m^ ± 2.37
PC	7.32^j^ ± 0.03	1.37^c^ ± 0.01	2.51^c^ ± 0.04	957.41^b^ ± 11.65	6.63 ×10^-1i^ ± 0.01	4.34^ij^ ± 0.11	0.92^c^ ± 0.01	77.95^k^ ± 0.52
T1	6.01^d^ ± 0.09	1.67^j^ ± 0.03	3.31^d^ ± 0.14	966.70^e^ ± 11.21	6.43 ×10^-1g^ ± 0.01	4.34^j^ ± 0.03	1.27^f^ ± 0.02	75.24^ij^ ± 0.23
T2	5.82^c^ ± 0.05	1.72^k^ ± 0.02	3.45^e^ ± 0.06	969.59^f^ ± 10.64	6.40 ×10^-1fg^ ± 0.01	4.28^h^ ± 0.06	1.39^h^ ± 0.01	74.13^hi^ ± 0.70
T3	5.45^b^ ± 0.03	1.84^l^ ± 0.02	3.33^d^ ± 0.25	963.62^d^ ± 10.64	6.53 ×10^-1h^ ± 0.01	4.34^j^ ± 0.12	1.13^d^ ± 0.02	76.09^j^ ± 0.85
T4	6.07^d^ ± 0.25	1.65^j^ ± 0.07	3.54^f^ ± 0.08	972.49^h^ ± 10.51	6.43 ×10^-1g^ ± 0.01	4.26^gh^ ± 0.05	1.44^i^ ± 0.03	73.5^0gh^ ± 0.53
T5	8.53^l^ ± 0.28	1.17^b^ ± 0.04	2.72^b^ ± 0.06	958.65^c^ ± 10.37	6.67 ×10^-1j^ ± 0.01	4.48^k^ ± 0.08	0.83^b^ ± 0.06	79.65^l^ ± 0.88
T6	6.64^f^ ± 0.08	1.51^h^ ± 0.02	4.86^j^ ± 0.05	989.51^l^ ± 10.39	6.13 ×10^-1cd^ ± 0.01	4.06^c^ ± 0.05	2.24^k^ ± 0.04	65.12^cd^ ± 0.81
T7	6.30^e^ ± 0.05	1.59^i^ ± 0.01	3.92^g^ ± 0.06	977.54^i^ ± 10.54	6.30 ×10^-1e^ ± 0.01	4.23^fg^ ± 0.03	1.61^g^ ± 0.06	70.50^f^ ± 1.79
T8	6.92^g^ ± 0.03	1.45^g^ ± 0.01	4.74^i^ ± 0.13	985.50^k^ ± 10.68	6.13 ×10^-1cd^ ± 0.01	4.18^e^ ± 0.05	2.03^j^ ± 0.06	67.69^e^ ± 1.29
T9	7.33^i^ ± 0.03	1.36^d^ ± 0.01	3.58^f^ ± 0.08	971.40^g^ ± 10.52	6.43 ×10^-1g^ ± 0.01	4.30^hi^ ± 0.02	1.41^e^ ± 0.03	72.31^g^ ± 1.95
T10	6.91^g^ ± 0.06	1.45^g^ ± 0.02	4.05^h^ ± 0.05	979.04^j^ ± 10.08	6.37 ×10^-1f^ ± 0.01	4.20^ef^ ± 0.02	1.74^i^ ± 0.03	69.21^e^ ± 0.99
T11	7.30^h^ ± 0.04	1.37^e^ ± 0.01	5.46^j^ ± 0.09	997.05^n^ ± 10.27	6.10 ×10^-1c^ ± 0.01	4.12^d^ ± 0.04	2.33^m^ ± 0.49	59.70^a^ ± 0.79
T12	6.88^g^ ± 0.12	1.45^g^ ± 0.03	5.05^j^ ± 0.05	992.32^m^ ± 10.59	6.17 ×10^-1d^ ± 0.01	4.14^d^ ± 0.04	2.34^l^ ± 0.03	66.24^de^ ± 1.14
T13	9.27^m^ ± 0.21	1.08^a^ ± 0.03	6.47^m^ ± 0.03	1015.87^r^ ± 10.15	5.87 ×10^-1a^ ± 0.01	3.87^a^ ± 0.08	3.52^p^ ± 0.04	59.47^a^ ± 1.07
T14	7.48^i^ ± 0.07	1.34^cd^ ± 0.02	5.57^k^ ± 0.06	1002.67^o^ ± 13.3	5.97 ×10^-1b^ ± 0.02	4.04c ± 0.07	2.74^n^ ± 0.03	63.70^bc^ ± 1.54
T15	8.65^k^ ± 0.11	1.15^b^ ± 0.02	5.87^k^ ± 0.04	1007.35^q^ ± 12.9	5.97 ×10^-1b^ ± 0.01	4.00^b^ ± 0.06	2.91^o^ ± 0.04	61.59^b^ ± 1.12
T16	7.24^h^ ± 0.07	1.38^f^ ± 0.01	5.64^l^ ± 0.03	1003.52^p^ ± 12.7	6.00 ×10^-1b^ ± 0.01	4.05^c^ ± 0.06	2.77^n^ ± 0.04	63.25^b^ ± 1.00

Mean ± SD; *n* = 3; Means with similar letter within the same column do not differ significantly (*p* ≤ 0.05), according to DMRT; Cp–Specific Heat Capacity; EC – electrical conductivity; λ–thermal conductivity; Ʈ–Shear Stress.

**Table 5:** The engineering properties of the watermelon juice at storage Day 10.

TC	EC (mS/cm)	Resistivity (Ω·m)	Viscosity (mPa·s)	Density (kg/m^3^)	λ (W/m·K)	Cp (kJ/kg·K)	Ʈ (Pa)	Dielectric constant
NC	6.92^a^ ± 0.07	1.45^m^ ± 0.02	3.21^a^ ± 0.02	895.43^a^ ± 11.23	6.59 ×10^-1k^ ± 0.00	4.56^l^ ± 0.04	0.75^a^ ± 0.03	78.53^m^ ± 1.39
PC	12.96^j^ ± 0.06	0.77^c^ ± 0.00	3.74^c^ ± 0.04	924.85^b^ ± 11.60	6.40 ×10^-1i^ ± 0.01	4.31^ij^ ± 0.11	1.15^c^ ± 0.03	71.94^k^ ± 0.77
T1	10.28^d^ ± 0.16	0.97^j^ ± 0.02	3.49^d^ ± 0.17	946.39^e^ ± 11.18	6.25 ×10^-1g^ ± 0.01	4.32^j^ ± 0.03	1.57^f^ ± 0.02	69.97^ij^ ± 0.22
T2	9.95^c^ ± 0.09	1.01^k^ ± 0.01	3.64^e^ ± 0.08	949.23^f^ ± 10.63	6.22 ×10^-1fg^ ± 0.01	4.26^h^ ± 0.07	1.73^h^ ± 0.01	68.94^hi^ ± 0.66
T3	9.31^b^ ± 0.05	1.08^l^ ± 0.01	3.37^d^ ± 0.11	943.38^d^ ± 10.63	6.36 ×10^-1h^ ± 0.01	4.32^j^ ± 0.12	1.38^d^ ± 0.04	71.43^j^ ± 1.54
T4	10.37^d^ ± 0.43	0.97^j^ ± 0.04	3.75^f^ ± 0.08	952.07^h^ ± 10.50	6.26 ×10^-1g^ ± 0.01	4.25^gh^ ± 0.04	1.78^i^ ± 0.04	68.46^gh^ ± 0.41
T5	14.58^l^ ± 0.48	0.69^b^ ± 0.02	2.85^b^ ± 0.08	938.52^c^ ± 10.36	6.49 ×10^-1j^ ± 0.01	4.45^k^ ± 0.08	0.78^b^ ± 0.06	74.20^l^ ± 0.95
T6	11.36^f^ ± 0.13	0.88^h^ ± 0.01	5.12^j^ ± 0.03	968.73^l^ ± 10.38	5.95 ×10^-1cd^ ± 0.01	4.04^c^ ± 0.05	2.13^k^ ± 0.03	60.56^cd^ ± 0.76
T7	10.78^e^ ± 0.08	0.93^i^ ± 0.01	4.16^g^ ± 0.07	957.01^i^ ± 10.53	6.12 ×10^-1e^ ± 0.01	4.21^fg^ ± 0.03	1.53^g^ ± 0.06	65.90^f^ ± 1.22
T8	11.82^g^ ± 0.05	0.85^g^ ± 0.01	4.99^i^ ± 0.09	964.81^k^ ± 10.67	5.96 ×10^-1cd^ ± 0.00	4.16^e^ ± 0.05	1.91^j^ ± 0.04	62.88^e^ ± 1.22
T9	12.54^i^ ± 0.04	0.80^d^ ± 0.01	3.73^f^ ± 0.05	951.01^g^ ± 10.51	6.25 ×10^-1g^ ± 0.01	4.28^hi^ ± 0.02	1.33^e^ ± 0.01	67.60^g^ ± 1.27
T10	11.71^g^ ± 0.28	0.86^g^ ± 0.02	4.30^h^ ± 0.06	958.48^j^ ± 10.08	6.18 ×10^-1f^ ± 0.01	4.18^ef^ ± 0.02	1.65^i^ ± 0.03	64.49^e^ ± 0.99
T11	11.99^h^ ± 0.18	0.83^e^ ± 0.02	5.00^j^ ± 0.05	987.18^n^ ± 10.27	5.95 ×10^-1c^ ± 0.01	4.09^d^ ± 0.02	2.44^m^ ± 0.03	57.10^a^ ± 0.47
T12	11.59^g^ ± 0.20	0.86^g^ ± 0.02	4.99^j^ ± 0.08	976.44^m^ ± 10.58	5.99 ×10^-1d^ ± 0.00	4.12^d^ ± 0.04	2.24^l^ ± 0.02	61.94^de^ ± 0.61
T13	14.60^m^ ± 0.26	0.68^a^ ± 0.02	5.68^m^ ± 0.03	1008.76^r^ ± 10.15	5.79 ×10^-1a^ ± 0.01	3.83^a^ ± 0.08	3.30^p^ ± 0.03	57.59^a^ ± 0.72
T14	12.51^i^ ± 0.12	0.80^cd^ ± 0.01	5.54^k^ ± 0.08	983.62^o^ ± 13.27	5.82 ×10^-1b^ ± 0.02	4.01^c^ ± 0.08	2.60^n^ ± 0.03	59.24^bc^ ± 1.43
T15	14.01^k^ ± 0.12	0.71^b^ ± 0.01	5.25^k^ ± 0.04	997.78^q^ ± 12.89	5.86 ×10^-1b^ ± 0.00	3.97^b^ ± 0.05	2.75^o^ ± 0.03	59.14^b^ ± 1.12
T16	12.10^h^ ± 0.12	0.83^f^ ± 0.01	5.63^l^ ± 0.04	984.45^p^ ± 12.66	5.87 ×10^-1b^ ± 0.01	4.02^c^ ± 0.07	2.64^n^ ± 0.03	58.95^b^ ± 0.73

Mean ± SD; *n* = 3; Means with similar letter within the same column do not differ significantly (*p* ≤ 0.05), according to DMRT; Cp–Specific Heat Capacity; EC – electrical conductivity; λ–thermal conductivity; Ʈ–Shear Stress.

The substantial increment observed in the viscosity, density, and shear stress results–primarily in the multi-additive treated samples (T11–T16), may be linked to the integration of honey, lime juice, and plant extracts into the watermelon juice. These additives have the potential to infuse natural sugars, polysaccharides, and other soluble solids into the juice, hence swiftly increasing its viscosity, density, and shear stress values (Abdullah et al., 2018; Bibi et al., 2025). Also, the decrease in the thermal conductivity, and specific heat capacity values exhibited by the fortified juice sample, may be linked to the phytochemicals, soluble sugars, and other soluble compounds present in the natural additives (Cruzalegui and Siche, 2025). It has been previously reported that polysaccharides and other soluble solids, have the ability to displace free water from the fruit juice, and thus lower its potential to transmit thermal energy within the matrix (Mwape et al., 2023; Assawarachan and Tantikul, 2025). Notably, the decline in the dielectric constant values in the enriched juice samples, suggests a reduction in free water activity in the juice matrix, which may be caused by the presence of nonpolar phytochemicals and other soluble compounds (Brglez Mojzer et al., 2016).

The stability observed in treated juice samples engineering properties evaluated, throughout the storage period, is a critical factor that enhances machine efficiency and preservation ability. For instance, electrical conductivity is one of the major factors to be considered in design of food processing systems, as it directly affects the ohmic heating efficacy. Juice specimens having greater electrical conductivity values, may tend to permit easy charged ions mobility, hence leading to accelerated and homogeneous thermal production (Assawarachan and Tantikul, 2025). Additionally, the smaller thermal conductivity and specific heat capacity heating and cooling rates of a fruit juice processing machine, hence lowering the amount of thermal energy needed to increase the system temperature. This is primarily this is due to the smaller specific heat capacity values of the treated juice samples (Zia et al., 2024). Furthermore, Goli et al. (2025) stated that elevated viscosity and density, is an indication of reduce sedimentation and phase separation rates, leading to a better approach for additive fortification in food processing. Subsequently, this may reduce energy consumption by decreasing heating duration, and optimizing energy utilization, as well as inhibiting microbial growth during juice preservation.

The increase in electrical conductivity and viscosity may tend to reduce the free water activity (hypertonic environment), and increase the electron mobility in the juice, hence creating osmotic problems, and facilitating the antimicrobial actions of the phytochemicals. According to Mari et al. (2024), osmotic imbalance and an ionized medium may compromise microbial cell membrane integrity, resulting in potential leakage of intracellular components and thereby reducing the antimicrobial effectiveness of the phytochemicals. This study advances natural preservation of watermelon juice by illustrating that the effects of treatments can be assessed concurrently across the evaluated parameters. Additionally, the established relationships offer an appropriate experimental basis for future artificial neural network modeling of specific responses. This study’s information highlights the usefulness of sustainable additives, as these data ranges serve as essential input data needed, for designing automated fruit juice pasteurization and handling systems.

### Microbiological evaluation

The results of the microbial stability of the juiced samples throughout the experimental period are presented in Tables 6, 7. It was noted that both storage duration and treatment concentration have a significant impact on the microbial stability of the juice samples. Notably, the treatment concentration plays a substantial role in inhibiting microbial proliferation, as the NC unit displayed the maximum microbial growth (TBC–29.60 × 10^4^ CFU/mL at Day 10, ToFC–14.79 × 10^3^ CFU/mL at Day 10), throughout the storage period. This swift increase observed in the juice samples during storage, particularly in the untreated sample, may be attributed to high moisture content, soluble sugars, dissolved organic solids, and a favorable pH level of the water melon juice, all of which have been proven to enhance microbial metabolism and proliferation (Possas et al., 2018; Lima et al., 2026). The smaller standard deviations values observed in certain microbial counts suggest minimal variability among replicates under specific treatment and storage conditions. Nonetheless, these data should not be regarded as indicative of biological homogeneity or as validation of a particular microbial or cellular pathway.

**Table 6:** Total bacterial count (×10^4^ CFU/mL).

Code	Day 0	Day 2	Day 4	Day 6	Day 8	Day 10
NC	0.86^o^ ± 0.04	2.60^o^ ± 0.15	5.41^o^ ± 0.15	10.45^o^ ± 0.29	16.98^o^ ± 0.23	29.60^o^ ± 0.55
PC	0.77^f^ ± 0.02	1.08^f^ ± 0.03	1.74^f^ ± 0.09	3.77^f^ ± 0.10	4.33^f^ ± 0.12	5.12^f^ ± 0.04
T1	0.81^l^ ± 0.02	1.51^l^ ± 0.06	2.59^l^ ± 0.05	4.84^l^ ± 0.06	6.34^l^ ± 0.16	7.79^l^ ± 0.05
T2	0.82^n^ ± 0.03	1.56^n^ ± 0.05	2.76^n^ ± 0.06	5.36^n^ ± 0.29	6.58^n^ ± 0.12	8.12^n^ ± 0.06
T3	0.77^k^ ± 0.02	1.33^k^ ± 0.03	2.36^k^ ± 0.05	4.58^k^ ± 0.21	5.76^k^ ± 0.04	7.18^k^ ± 0.05
T4	0.80^m^ ± 0.01	1.49^m^ ± 0.06	2.67^m^ ± 0.04	4.95^m^ ± 0.04	6.56^m^ ± 0.37	7.94^m^ ± 0.05
T5	0.74^h^ ± 0.02	1.18^h^ ± 0.05	1.98^h^ ± 0.05	3.97^h^ ± 0.06	4.65^h^ ± 0.22	5.73^h^ ± 0.03
T6	0.71^h^ ± 0.02	1.02^h^ ± 0.06	1.80^h^ ± 0.06	3.69^h^ ± 0.05	4.28^h^ ± 0.08	6.94^h^ ± 0.04
T7	0.77^j^ ± 0.02	1.30^j^ ± 0.04	2.34^j^ ± 0.10	4.44^j^ ± 0.04	5.54^j^ ± 0.13	6.94^j^ ± 0.09
T8	0.72^g^ ± 0.01	1.14^g^ ± 0.07	1.95^g^ ± 0.04	3.89^g^ ± 0.08	4.62^g^ ± 0.07	5.45^g^ ± 0.07
T9	0.74^i^ ± 0.01	1.20^i^ ± 0.02	2.10^i^ ± 0.04	4.17^i^ ± 0.15	4.89^i^ ± 0.06	5.87^i^ ± 0.05
T10	0.78^k^ ± 0.02	1.34^k^ ± 0.04	2.39^k^ ± 0.06	4.62^k^ ± 0.12	5.81^k^ ± 0.16	6.95^k^ ± 0.06
T11	0.66^cd^ ± 0.02	0.88^cd^ ± 0.05	1.62^cd^ ± 0.04	3.54^cd^ ± 0.18	3.84^cd^ ± 0.07	4.35^cd^ ± 0.11
T12	0.68^e^ ± 0.02	0.95^e^ ± 0.03	1.71^e^ ± 0.05	3.51^e^ ± 0.05	4.07^e^ ± 0.05	5.01^e^ ± 0.03
T13	0.59^a^ ± 0.02	0.73^a^ ± 0.04	1.31^a^ ± 0.03	2.85^a^ ± 0.04	3.12^a^ ± 0.04	3.29^a^ ± 0.06
T14	0.64^c^ ± 0.02	0.87^c^ ± 0.03	1.59^c^ ± 0.08	3.30^c^ ± 0.04	3.80^c^ ± 0.04	4.34^c^ ± 0.04
T15	0.60^b^ ± 0.03	0.78^b^ ± 0.03	1.37^b^ ± 0.07	3.00^b^ ± 0.07	3.41^b^ ± 0.07	3.63^b^ ± 0.04
T16	0.66^de^ ± 0.03	0.90^de^ ± 0.03	1.63^de^ ± 0.03	3.41^de^ ± 0.04	3.95^de^ ± 0.04	4.86^de^ ± 0.09

Mean ± SD; *n* = 3; Means with similar letter within the same column do not differ significantly (*p* ≤ 0.05), according to DMRT.

**Table 7:** Total fungal count (×10^3^ CFU/mL).

Code	Day 0	Day 2	Day 4	Day 6	Day 8	Day 10
NC	0.66^l^ ± 0.04	2.41^l^ ± 0.13	4.02^l^ ± 0.08	6.29^l^ ± 0.06	9.68^l^ ± 0.05	14.79^l^ ± 0.21
PC	0.57^f^ ± 0.02	1.63^f^ ± 0.03	1.99^f^ ± 0.02	2.59^f^ ± 0.03	3.04^f^ ± 0.57	3.08^f^ ± 0.06
T1	0.61^j^ ± 0.03	1.82j ± 0.06	2.37^j^ ± 0.04	2.42_j_ ± 0.03	3.47^j^ ± 0.06	4.52^j^ ± 0.08
T2	0.62^k^ ± 0.01	1.87^k^ ± 0.03	2.42^k^ ± 0.02	2.53^k^ ± 0.02	3.58^k^ ± 0.04	4.67^k^ ± 0.05
T3	0.58^i^ ± 0.03	1.76^i^ ± 0.03	2.26^i^ ± 0.03	2.30^i^ ± 0.12	3.20^i^ ± 0.02	4.27^i^ ± 0.04
T4	0.60^k^ ± 0.01	1.87^k^ ± 0.04	2.39^k^ ± 0.04	2.46^k^ ± 0.04	3.57^k^ ± 0.05	4.62^k^ ± 0.04
T5	0.56^g^ ± 0.02	1.69^g^ ± 0.04	2.12^g^ ± 0.04	1.95^g^ ± 0.04	2.77^g^ ± 0.06	3.54^g^ ± 0.03
T6	0.52^e^ ± 0.03	1.55^e^ ± 0.04	1.93^e^ ± 0.03	2.15^e^ ± 0.03	2.30^e^ ± 0.04	2.87^e^ ± 0.06
T7	0.57^h^ ± 0.02	1.74^h^ ± 0.03	2.18^h^ ± 0.03	2.18^h^ ± 0.03	3.09^h^ ± 0.04	4.05^h^ ± 0.05
T8	0.56^f^ ± 0.02	1.67^f^ ± 0.09	2.05^f^ ± 0.06	1.88^f^ ± 0.03	2.58^f^ ± 0.03	3.34^f^ ± 0.04
T9	0.61^g^ ± 0.02	1.69^g^ ± 0.03	2.09^g^ ± 0.02	1.95^g^ ± 0.05	2.77^g^ ± 0.03	3.57^g^ ± 0.06
T10	0.60^i^ ± 0.03	1.78^i^ ± 0.05	2.26^i^ ± 0.04	2.25^i^ ± 0.04	3.20^i^ ± 0.05	4.20^i^ ± 0.05
T11	0.52^c^ ± 0.03	1.51^c^ ± 0.05	1.82^c^ ± 0.05	1.96^c^ ± 0.02	2.16^c^ ± 0.03	2.72^c^ ± 0.03
T12	0.53d^e^ ± 0.02	1.57^de^ ± 0.03	1.89^de^ ± 0.05	1.66^de^ ± 0.02	2.24^de^ ± 0.04	2.98^de^ ± 0.04
T13	0.47^a^ ± 0.02	1.45^a^ ± 0.04	1.71^a^ ± 0.03	1.79^a^ ± 0.03	1.81^a^ ± 0.07	2.17^a^ ± 0.04
T14	0.52^cd^ ± 0.03	1.55^cd^ ± 0.03	1.90^cd^ ± 0.06	1.58^cd^ ± 0.04	2.17^cd^ ± 0.05	2.75^cd^ ± 0.04
T15	0.47^b^ ± 0.02	1.45^b^ ± 0.04	1.75^b^ ± 0.04	1.47^b^ ± 0.04	2.04^b^ ± 0.04	2.31^b^ ± 0.06
T16	0.51^de^ ± 0.04	1.55^de^ ± 0.03	1.87^de^ ± 0.06	1.63d^e^ ± 0.03	2.26^de^ ± 0.06	2.87^de^ ± 0.05

Mean ± SD; *n* = 3; Means with similar letter within the same column do not differ significantly (*p* ≤ 0.05), according to DMRT.

The results further affirm previous reports on the antimicrobial actions of sodium benzoate, even at lower concentrations (Chen and Zhong, 2018). As seen in the results, the higher acidity (lower pH values) of the lime juice, created an acidic condition that enhances the ionic mobility and electrical conductivity, of the lime juice-based combination treatments (mainly T13, T15, and T11). This increased mobility and acidic environment, may corrode the microbial cell’s protective membrane, leading to leakage of intracellular materials, osmotic imbalance, and suppression of metabolic activities in the microorganisms. Furthermore, acidification may enhance the effectiveness of antimicrobial phytochemicals by increasing membrane permeability. Eventually, these actions may lead to increased microbial mortality (Penniston et al., 2008; Rahmiati et al., 2024). Additionally, the honey will play a mild antimicrobial role by reducing the water activity level of the treated juice sample, while its organic acids and flavonoids contents will impair microbial growth and other metabolic activities (Eryılmaz et al., 2026).

Generally, the results indicate that the one-component treatments display moderate preservation ability, while treatments containing lime juice and honey tend to exhibit higher preservation ability than those with only CSE, CiE, BLE, and GRE. This highlights the significance of lower acidity (caused by the lime juice) and osmotic inhibition (caused by the honey) in food preservation. The progressive increase in TBC and ToFC den noted in the treated samples during storage, indicates that, the antimicrobial efficacy of the natural additives was inadequate to suppress bacterial and fungal growth over the 10-day observation period. The detected alterations may indicate progressive deterioration or conversion of bioactive phytochemicals and modifications in the physicochemical conditions over storage time (Papadochristopoulos et al., 2021; Rathee et al., 2023). According to Rathee et al. (2023), polyphenols are prone to rapid oxidation in the presence of UV light and high temperature, leading to a gradual reduction in their antimicrobial properties. Remarkably, the closely clustered recordings in the TBC and ToFC during T11 to T16 may be linked to the proximity of the treatments’ pH values, antioxidant profiles (Tables 3–5), and the potential comparability, and compatibility of their bioactive compounds. Since the current investigation measured overall microbial load rather than specific microbial taxa or community structure, the findings do not reveal alterations in particular bacterial or fungal species or elucidate organism-specific or cellular mechanisms. Therefore, the antimicrobial patterns, such as treatment- or storage-induced effects on microbial proliferation observed in this study, should be considered plausible interpretations rather than definitively established mechanisms.

Among the evaluated formulations, T13 consistently demonstrated the minimal bacterial and fungal loads throughout the 10-day storage duration, reflecting enhanced microbial preservation efficacy *in vitro*. This attribute of the T13 treatment can be linked to the strong antimicrobial actions of eugenol, cinnamaldehyde, terpenoids, and gingerol, which are the major phytochemicals present in the clove, cinnamon, bay leaf, and ginger rhizome extracts used in the treatment formulation. Eugenol has the ability to compromise microbial cell membrane integrity and metabolic processes (Fajdek-Bieda et al., 2025); cinnamaldehyde has high efficacy in inhibiting ATP production, enzymatic reactions (mainly cell wall synthesis), and cell membrane function, leading to the leakage of essential intracellular contents (Vasconcelos et al., 2018); terpenoids and gingerol have the potential to impair oxidative and metabolic processes in both bacterial and fungal cells, leading to decreased microbial energy production and impaired protein synthesis (Zhang et al., 2023). The mechanistic actions of these essential phytochemicals may result in poor microbial activity, and eventually lead to microbial cell lysis. This study’s findings aligned with previous reports on watermelon juice storability, conducted by Liu et al. (2026).

These findings have highlighted the feasibility of employing eco-friendly natural bioadditives as prospective antimicrobial agents for watermelon juice. Additionally, the results revealed that, the natural additives investigated in this research, can facilitate the design of reliable fruit juice preservation systems. Primarily, the ability of the biopreservatives to impair microbial proliferation (enhanced microbial stability), may substantially assist in the development of automated monitoring frameworks, biosensors, and forecasting models. The rapid microbial growth observed in some of the juice samples, could be responsible for the deterioration in the thermo-rheological and electrochemical properties, of the watermelon juice during storage (Table 5). It has been reported that microbial activity, can substantially alter the engineering properties of fruit juices, through metabolic reactions such as oxidation and ionic changes (Mundanat et al., 2025).

### Regression analysis of the treatment and storage effects, on the engineering properties

Tables 8, 9 present the results of the linear regression analysis of the impact of treatment and storage duration, on the watermelon juice engineering properties. Based on the treatment factor, ɛ, λ, τ, and η displayed strong relationships (R ≥ 0.820) with the treatment, and the *R*^2^ values suggesting that about 65 to 72% of the parameters variability, can be attributed to the treatment composition and concentration. The elevated positive β results recorded in the case of ρ, τ, and η, are an indication that treatments formulated from natural additives, can substantially enhance the juice’s mechanical and rheological properties. Contrariwise, the documented negative β results for λ, ɛ, Cp, and ρ indicate that, increasing treatment concentrations leads to a decline in these parameters. This reduction may be caused by a reduction in free-water activity, and a concurrent increase in soluble compounds, within the juice sample as reported by (Assawarachan and Tantikul, 2025).

**Table 8:** Linear regression of the essential parameters as a Function of treatment.

Parameter	*R*	*R* ^2^	Adjusted *R*^2^	Std. error of the estimate	Standardized regression coefficient *β*	*p*-value
Electrical conductivity	0.416a	0.173	0.170	1.936	0.416	< 0.001^a^
Resistivity	0.478^a^	0.229	0.226	0.282	−0.478	< 0.001^a^
Viscosity	0.846^a^	0.716	0.715	0.598	0.846	< 0.001^a^
Density	0.844^a^	0.713	0.712	12.359	0.844	< 0.001^a^
Thermal conductivity	0.806^a^	0.649	0.648	0.015	−0.806	< 0.001^a^
Specific heat capacity	0.781^a^	0.610	0.609	0.114	−0.781	< 0.001^a^
Shear stress	0.822^a^	0.675	0.674	0.398	0.822	< 0.001^a^
Dielectric constant	0.820^a^	0.673	0.672	4.016	−0.820	< 0.001^a^

The superscript “a” indicates Predictor: (Constant) Treatment, correlation coefficient (*R*), coefficient of determination (*R*^2^).

**Table 9:** Linear regression of quality parameters as a function of storage duration.

Parameter	*R*	*R* ^2^	Adjusted *R*^2^	Std. error of the estimate	Standardized regression coefficient *β*	*p*-value
Electrical conductivity	0.711^a^	0.505	0.504	1.497	0.711	< 0.001^a^
Resistivity	0.631^a^	0.398	0.396	0.249	−0.631	< 0.001^a^
Viscosity	0.008^a^	6.91E-05	−0.003	1.123	0.008	0.881
Density	0.291^a^	0.085	0.082	22.059	−0.291	< 0.001^a^
Thermal conductivity	0.214^a^	0.046	0.043	0.0261	−0.0214	< 0.001^a^
Specific heat capacity	0.059^a^	3.43E-03	3.33E-04	0.182	−0.059	0^.^293
Shear stress	0.007^a^	5.508E-05	−3.059E-04	0.699	−0.007	0.894
Dielectric constant	0.203^a^	0.041	0.038	6.871	−0.203	< 0.001^a^

The superscript “a” indicates Predictor: (Constant) storage, correlation coefficient (*R*), coefficient of determination (*R*^2^).

Based on the storage factor (Table 9), although storage duration has a significant influence on most of the properties investigated, η, Cp, and τ were not significantly affected by the storage period (*p* < 0.001). The results suggested that the EC has a strong relation with storage duration (R = 0.711), and about 50.5% of the EC variability can be linked to storage duration. On the contrary, η and τ coefficient of determination values suggested that, these two properties showed minimal variability with storage period, with their changes mainly attributable to the treatment applied. Similarly, the non-significant *p*-values recorded for η, Cp, and τ during storage, imply that the juice’s rheological and heat storage behaviors, remained stable throughout the storage period. This stability may be linked to the applied treatment.

Generally, the regression results showed that storage duration primarily affected the microbial activities, and electrical properties of the fortified juice samples. However, most of the thermo-rheological properties were mainly influenced, by the applied treatment. Interestingly, the regression analysis established data-driven relationships between treatment and storage variables among the measured engineering and microbial features, offering an interpretable statistical foundation for their integration into the ANN modeling framework.

### The combined performance indices of the fortified watermelon juice samples

The results of the calculated NPBI, CI, MIE and SI values are presented in Table 10. The results revealed that, the multi-ingredient formulations predominantly displayed advantageous nutritional profiles, antioxidant activity, and microbial preservation compared to the baseline treatments. The optimized performance recorded in T13 and T15 samples, further affirmed the possibility of complementary mechanisms, among the essential compounds of the diverse components used in the treatment formulation. Hence, the evaluated efficacy of the integrated formulations indicates enhanced stability or additive effects, rather than definitive synergistic interactions. As already mentioned in previous studies, the natural additives used in the formulation of hybridized treatments have abundant phenolic compounds, flavonoids, and organic acids, and these compounds, coupled with the osmotic stress effects originating from honey. These bioactive compounds may have complementary antimicrobial actions by mechanisms like compromising microbial cellular integrity, shifting pH levels, and inducing osmotic stress (Eryılmaz et al., 2026).

**Table 10:** The calculated NPBI, CI, MIE, and SI values of the watermelon juice samples.

Treatment	NPBI	MACI	MIE at Day 10 (%)	SI
NC	1.00	ND	ND	ND
PC	1.04	ND	82.53	0.93
T1	2.27	1.00	73.48	0.83
T2	2.08	0.92	72.37	0.82
T3	1.90	0.83	75.52	0.85
T4	2.03	0.89	72.97	0.82
T5	1.88	0.77	80.42	0.91
T6	1.89	0.81	76.75	0.87
T7	2.57	1.16	76.37	0.86
T8	2.38	1.08	81.39	0.92
T9	2.43	1.02	79.96	0.90
T10	2.50	1.12	76.29	0.86
T11	3.01	1.37	85.13	0.96
T12	2.84	1.31	82.92	0.93
T13	3.85	1.73	88.72	1.00
T14	3.15	1.43	85.15	0.96
T15	3.63	1.61	87.58	0.99
T16	3.20	1.47	83.44	0.94

ND, not determined; the results in the table are calculated values, therefore no replication.

The results affirmed that the multi-component formulations showed the best performance. The computed NPBI and MACI metrics offer relative insights into dietary, phytochemicals, and antioxidant activities, with T13 and T15 demonstrating enhanced nutrient bioavailability (NPBI ≥ 3.50). Remarkably, the performance of the treatments, as indicated by the calculated indices, followed this ranking pattern: T13 > T15 > T11 ≈ T14 > T12 ≈ PC > T8 > T5 > T9 > T6 > T7 ≈ T10 > T3 > T1 > T2 ≈ T4which further clarifies the antioxidant and antimicrobial actions, of the results presented in Tables 3–5. This ranking offers a comparative overview of the nutritional, antioxidant, and microbial preservation efficacy of the formulations and endorses the relatively superior performance of T13 and T15 under the experimental conditions.

### ANN projection of engineering properties and microbial responses

Figures 1–11 and Supplementary Figures S1–S20 present the ANN results obtained for electrical conductivity, viscosity, total bacterial count, and total fungal count. The artificial neural network outcomes demonstrate that the treatment formulation and storage duration embedded adequate information within the experimental dataset to develop predictive models for the chosen engineering and microbial responses. Figure 1 shows the ANN model architecture for EC, while Figure 2 presents the regression curve fit for training, validation, and testing of the EC. The EC model demonstrated a high correlation between actual and estimated outputs across different data partitions, suggesting that the chosen neural network architecture effectively captured nonlinear relationships in the data repository.

**Figure 1:**
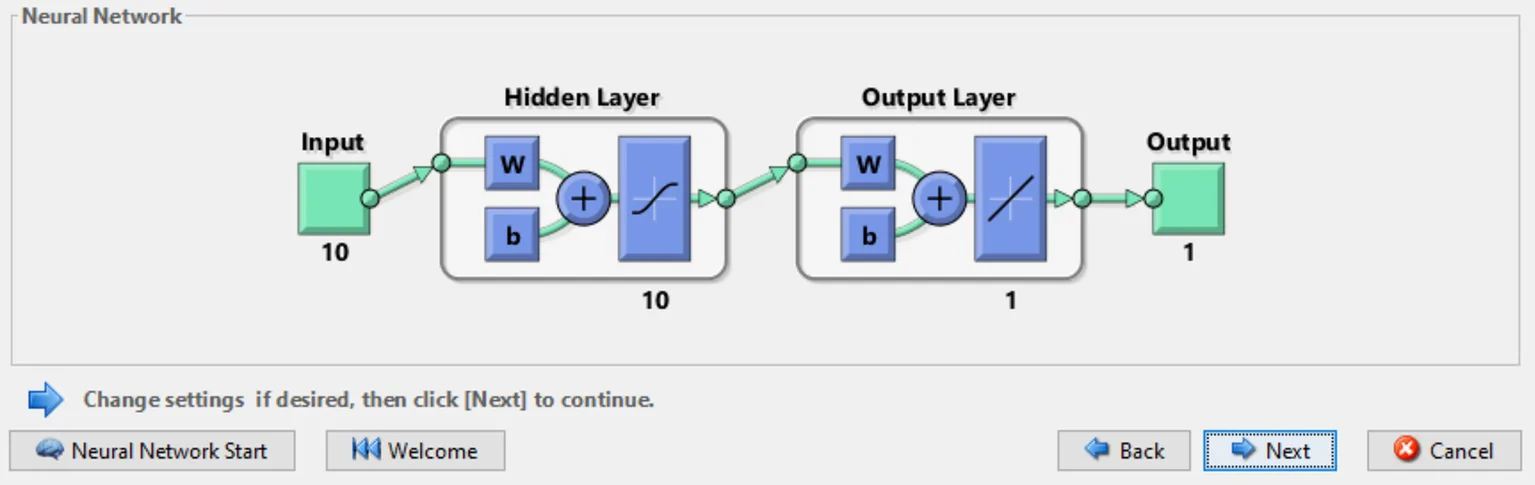
ANN model architecture for electrical conductivity.

**Figure 2:**
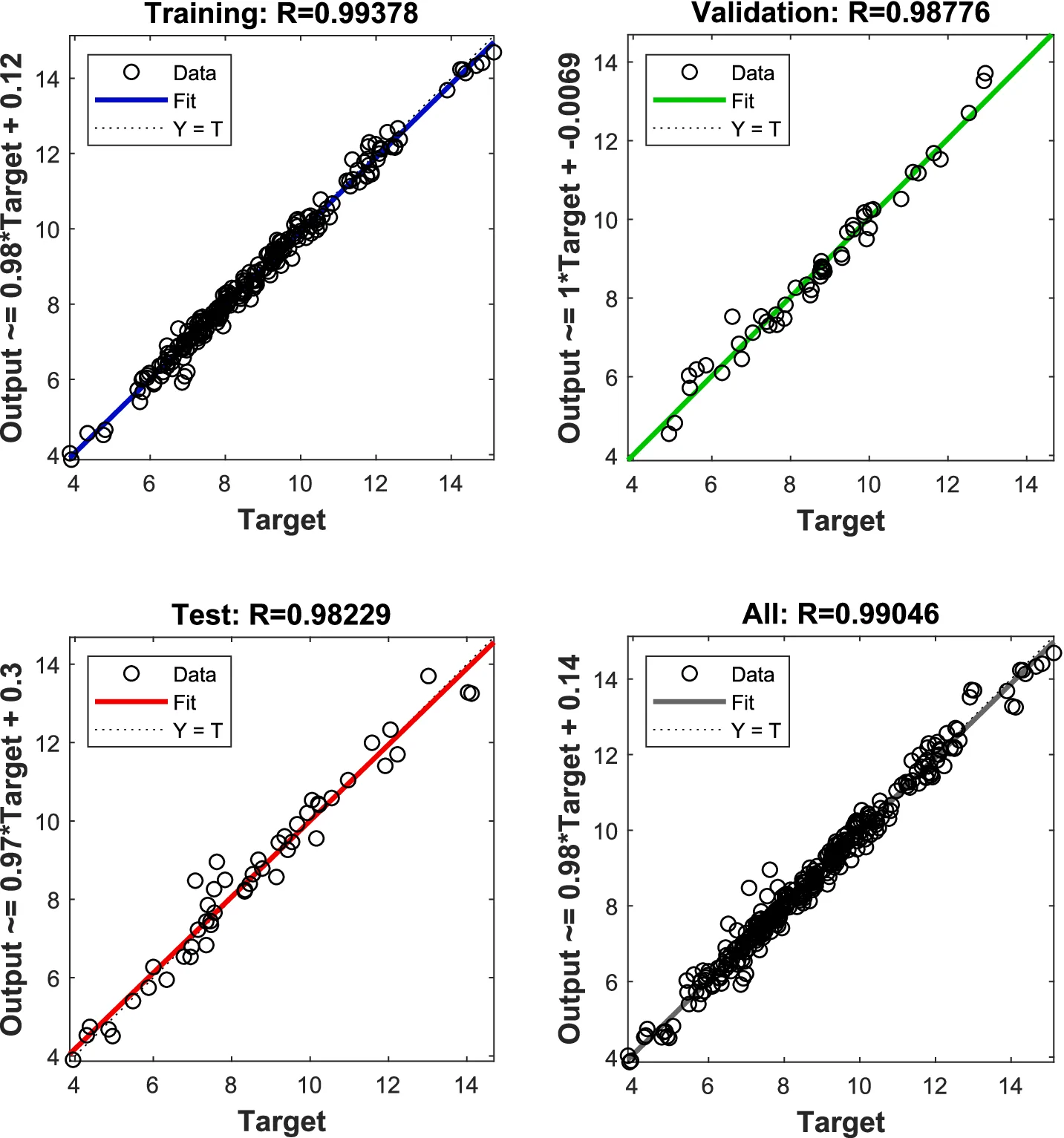
Model regression curve fit for training, validation and testing for electrical conductivity.

**Figure 3:**
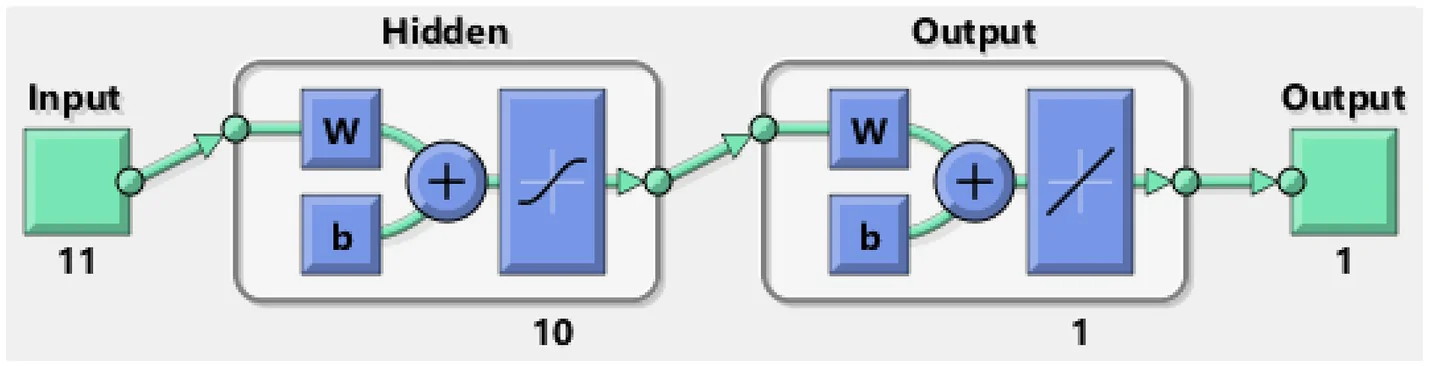
ANN model architecture for viscosity.

**Figure 4:**
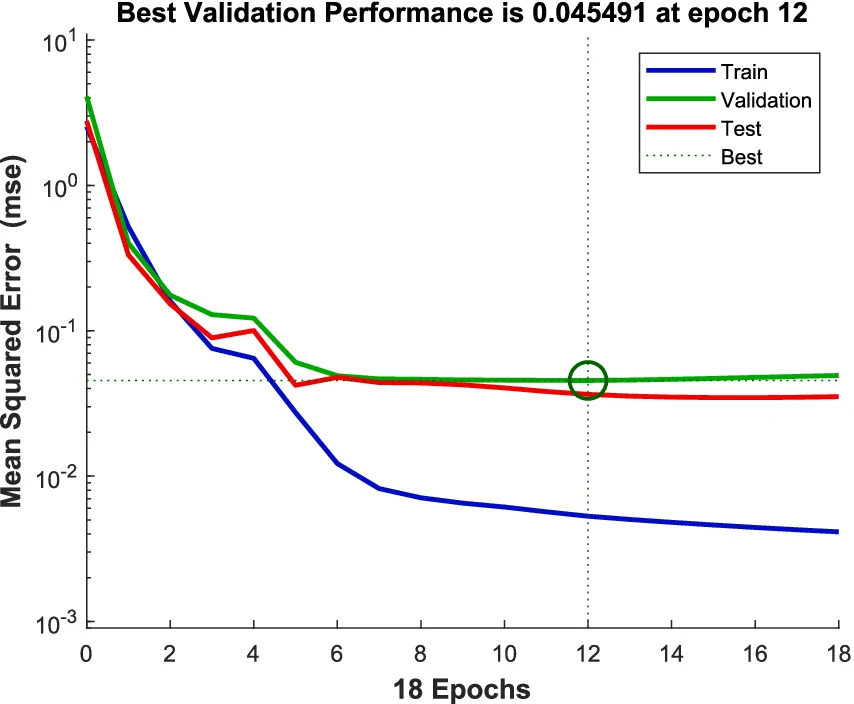
Model performance during training, validation and testing for viscosity.

**Figure 5:**
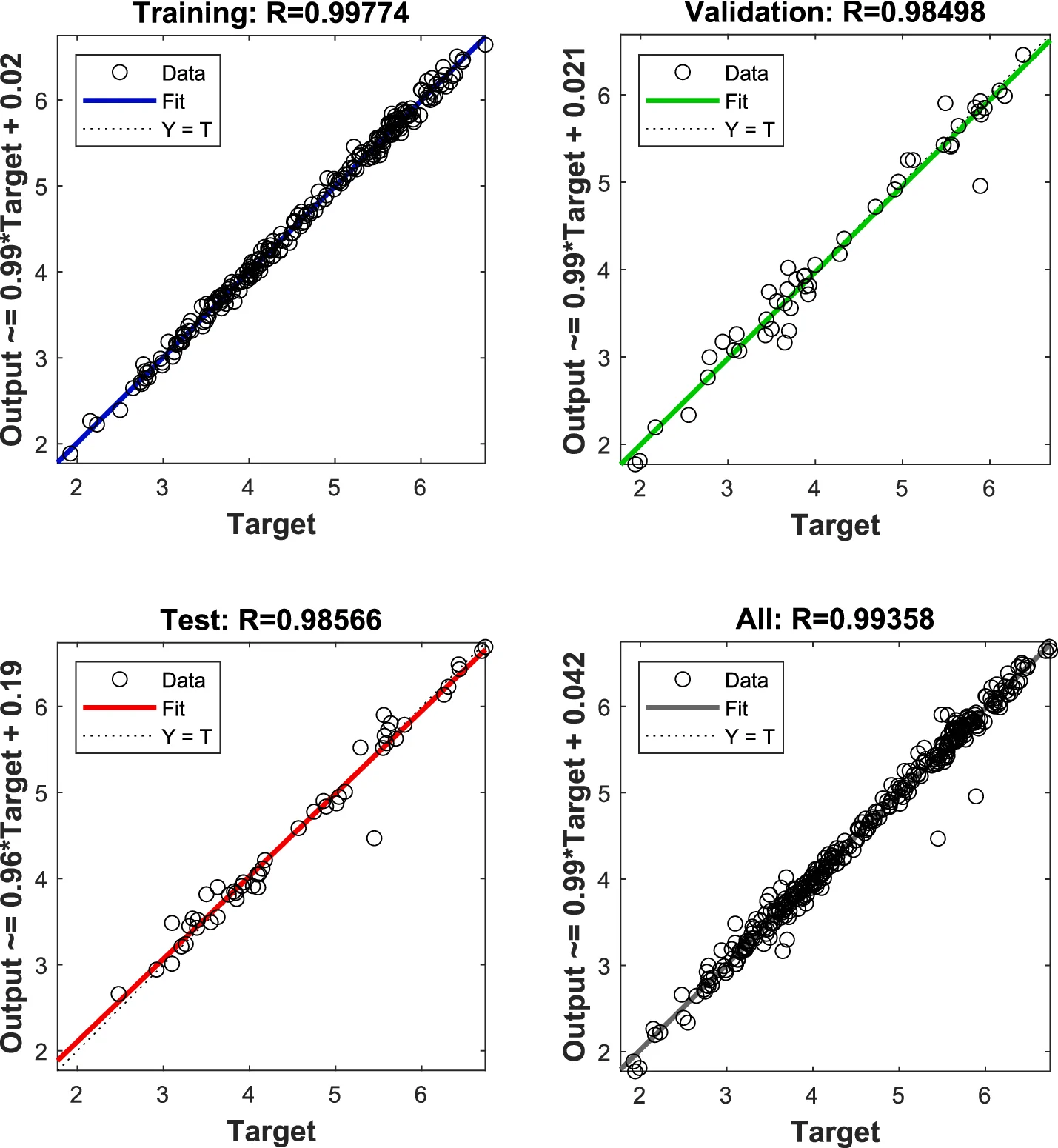
Model regression curve fit for training, validation and testing for viscosity.

**Figure 6:**
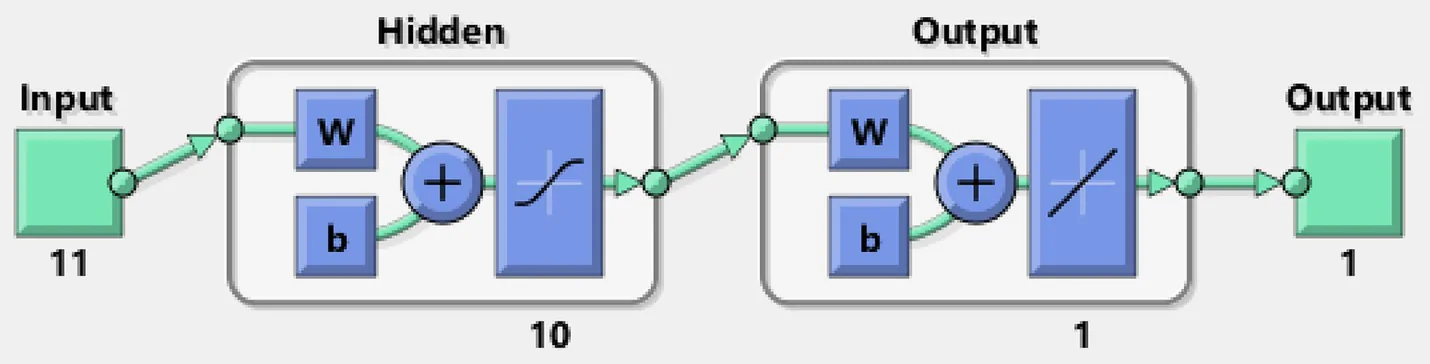
ANN Model architecture for TBC.

**Figure 7:**
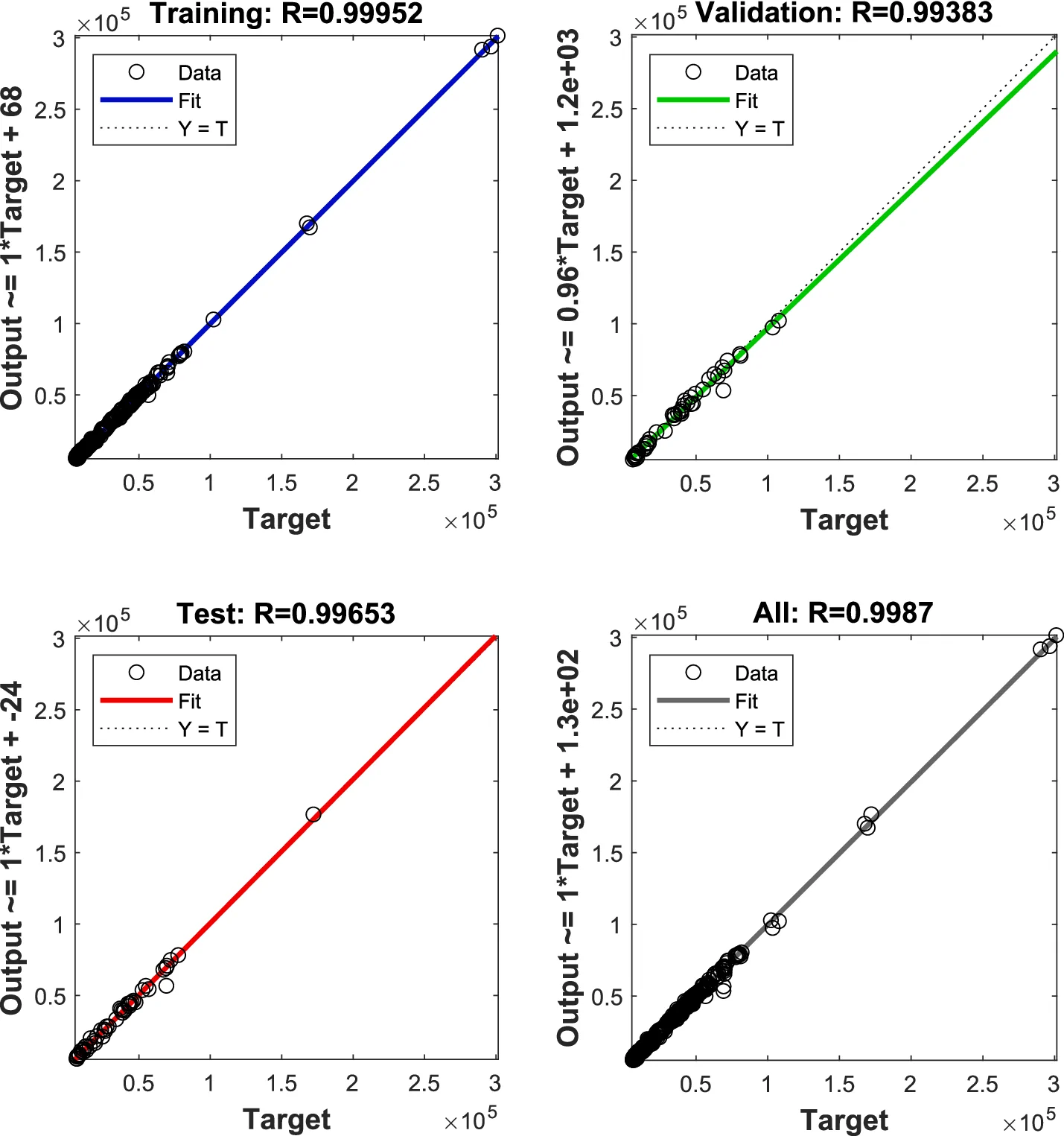
Model regression curve fit for training, validation and testing for TBC.

**Figure 8:**
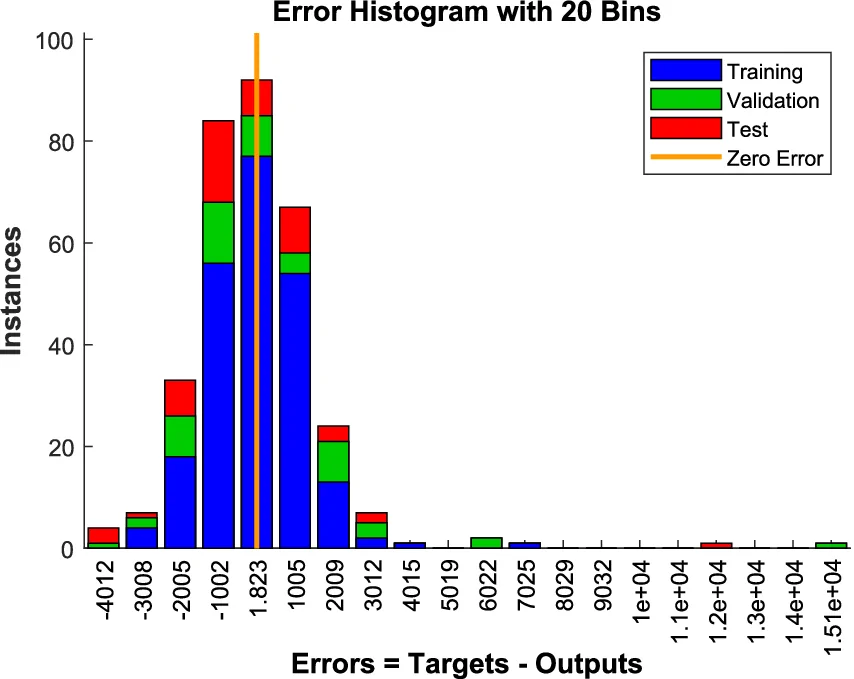
Model error histogram during training, validation and testing for TBC.

**Figure 9:**
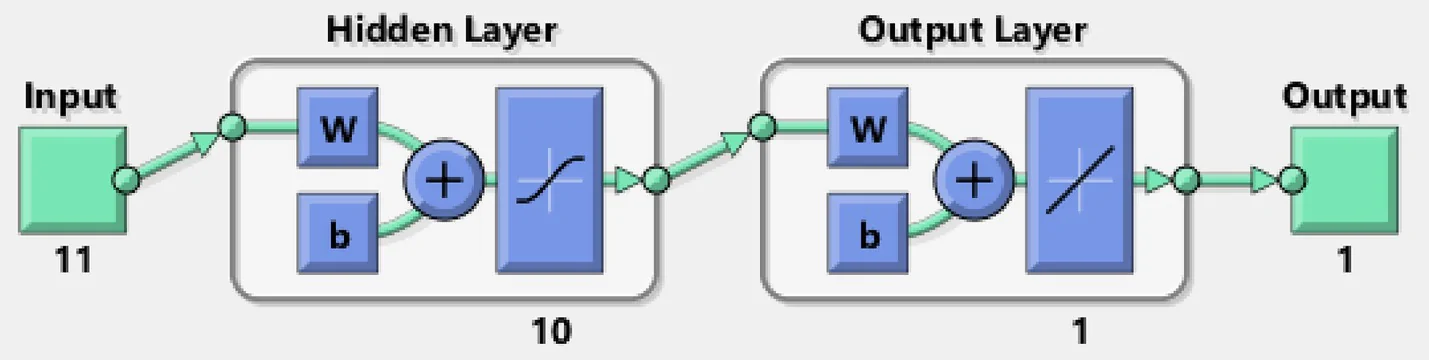
ANN model architecture for total fungal count.

**Figure 10:**
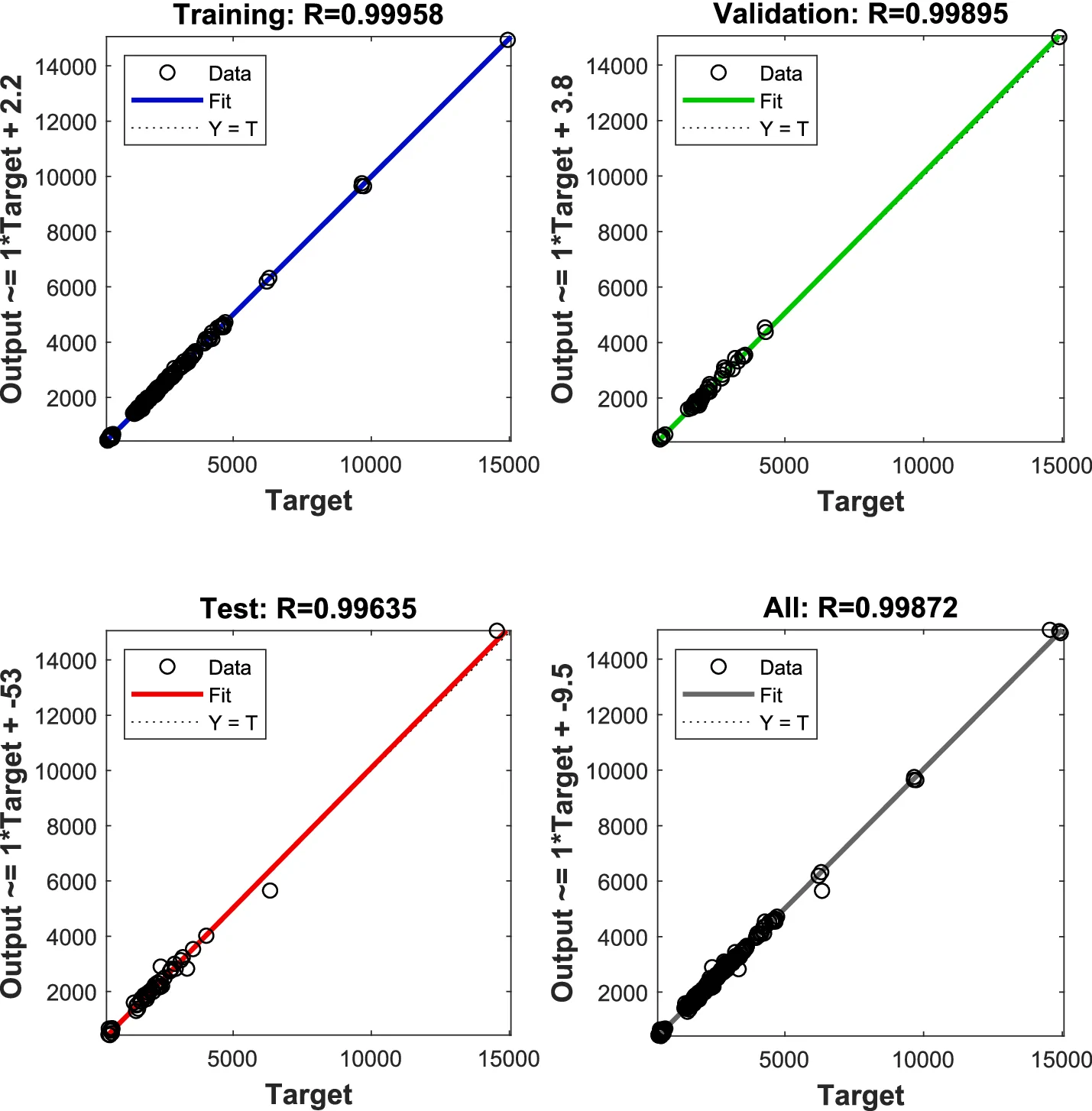
Model regression curve fit for training, validation and testing for total fungal count.

**Figure 11:**
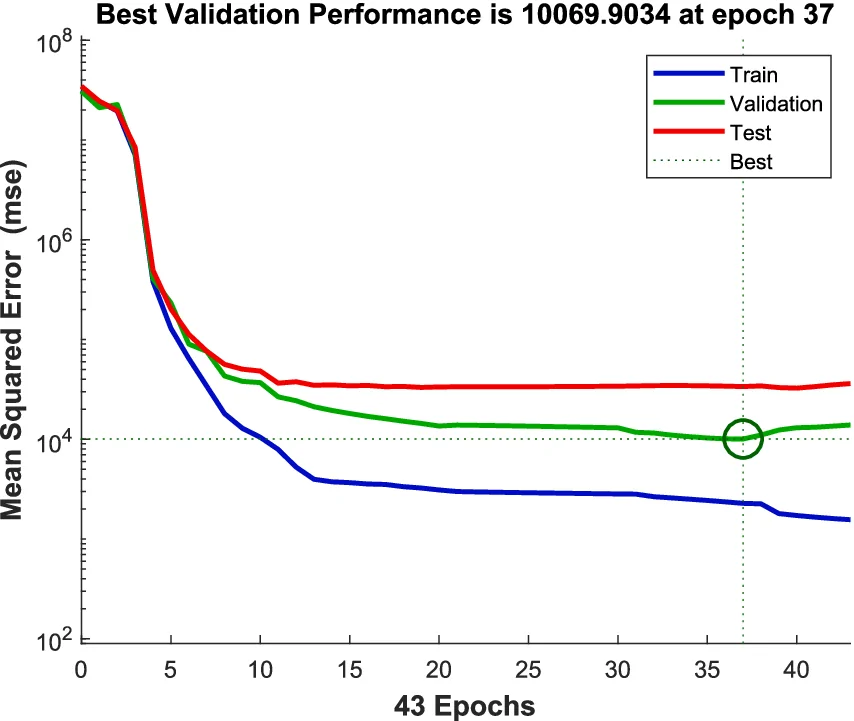
Model performance during training, validation and testing for total fungal count.

Figure 2 compares the targets against the output values. The high R-value achieved by the EC model signifies a robust alignment between the actual and neural network-predicted outputs within the tested dataset. Supplementary Figures S1–S6 further affirmed the reliability of the ANN, in modeling electrical conductivity during validation and testing. As previously mentioned in this study, reports from earlier studies suggest that elevated electrical conductivity in the treated juice, may be caused by the higher acidity level resulting from the incorporation of lime juice, and by the antimicrobial actions of phytochemicals found in the additives (Liu et al., 2026). Subsequently, the neural network effectively learned these relationships, resulting in high prediction accuracy.

Similarly, Figures 3–5 show: the viscosity ANN model architecture; model evaluation metrics across the training, validation, and testing phases for viscosity; and the model regression curve fit for training, validation, and testing for viscosity, respectively. While Supplementary Figures S1–S11, present: the ANN model training Set-up for viscosity; model training progress for viscosity; model train results in training, validation and testing for viscosity; model condition during training, validation, and testing; and prediction error histogram, respectively. As seen in Figure 3, the neural network model has the potential to learn the viscosity patterns from the provided input variables. The viscosity regression curve (Figure 4), revealed that the data training resulted in a high correlation coefficient (99.77%), mainly between the laboratory data and projected viscosity. The validation results demonstrated a strong correlation between actual and predicted viscosity values, reflecting reliable predictive performance on the validation dataset. The small MSE value (0.045491) document for viscosity indicates that, the model successfully reduced training error. These outcomes suggest that the neural network modeled the relationship between input features and viscosity in the training data; validation with unseen external datasets would be necessary to confirm its applicability to new juice samples.

Figures 6–8 present: the ANN model architecture for TBC; model regression curve fit for training, validation and testing for TBC; and model error histogram during training, validation and testing for TBC, respectively. Then, Supplementary Figures S12–S15, present: the TBC ANN model training Set-up; model training progress for TBC; TBC model state during training, validation and testing; and TBC model regression curve fit for training, validation and testing. The ANN regression outcomes demonstrated a high alignment between actual and estimated bacterial counts, reflecting that the model effectively learned the primary patterns in the bacterial population dynamics within the experimental dataset.

Also, as shown in Figure 8, the residual error distribution centered around zero, indicating strong alignment between actual and predicted TBC values, across all evaluated data partitions. Nevertheless, these outcomes should be regarded as indicative of the model’s internal performance rather than proof of generalization across separate datasets. Figures 9–11 show: the ToFC ANN model architecture; ToFC model regression curve fit for training, validation and testing; and ToFC model performance during training, validation and testing, respectively. While Supplementary Figures S16–S20 show: ToFC ANN Model Training Set-up; ToFC model training progress; ToFc model train results in training, validation and testing; ToFC status during training, validation and testing; and ToFC model error histogram during training, validation and testing, respectively. The ToFC ANN model demonstrated high concordance between actual and predicted outputs, with correlation coefficients of 0.99958, 0.99895, and 0.99635 for the training, validation, and testing datasets, respectively, and 0.99872 across the entire dataset. Additionally, the model performance during training showed that the neural network trained through epoch 37, whereupon it attained its minimum authentication loss. These findings demonstrate high model consistency within the experimental results.

The strong prediction results documented in this research highlighted that the chosen neural network architecture effectively modeled the nonlinear relationships between input features and fungal counts in the dataset (Taiwo et al., 2024,). Likewise, the constantly elevated R values (R ≥ 0.993) documented, is a suggestion that the ANN models effectively apprehended the nonlinear patterns inherent in the actual data, which is consistent with the earlier findings of Dehdashti et al. (2026). The outstanding ANN performances, regarding microbial activities (TBC prediction and ToFC prediction) demonstrated that, bacterial and fungal proliferations were highly anticipated, by primarily using treatment options and storage period as input layers. These outstanding projections for microbial growth, further affirmed the similar route of quality alteration taken, by both engineering and microbiological parameters, in the blended watermelon juice samples. The ANN models exhibited high concordance between actual and predicted outputs across the training, validation, and testing datasets, demonstrating their capacity to model complex nonlinear relationships within the experimental data. These findings validate the application of ANN as a supplementary modeling technique, for the analyzed engineering and microbial data, though additional independent datasets are necessary to confirm wider predictive applicability.

The results from the neural network indicate the value of data-centric modeling techniques for capturing nonlinear patterns in the dataset, which can be used for juice quality control, storability forecasting, and process enhancement during storage. This aligned to the previous findings of these authors (Shehzad, 2025; Warade, 2025). The prediction of thermo-rheological properties provide foundational data for subsequent system simulation of processing workflows, even though current experimental dataset does not demonstrate direct improvements in computational efficiency, system reliability, or energy consumption (Elkatatny, 2019). Furthermore, the precise projection of electrical conductivity obtained in this study, could serve as a foundation for subsequent research into data-driven surveillance techniques for beverage quality; however, the implementation and testing of a real-time biosensor system were outside the scope of the current research.

Table 11 summarizes the predictive accuracy of the ANN models for EC, viscosity, TBC, and ToFC. The models exhibited a strong correlation between actual and predicted values, as reflected by the elevated *R*^2^ scores and comparatively low RMSE values. Additionally, Table 12 shows a consistent performance gap between classical multiple linear regression and ANN models across the evaluated engineering and microbial responses. The constantly higher *R*^2^ and lower RMSE achieved by the ANN across all four outputs may be attributed to its capacity to model complex and potentially nonlinear relationships among the input features. Traditional linear regression models operate under the assumption of a linear, additive function between input variables and output unless explicitly extended with feature interactions or nonlinear basis functions. Generally, the comparative results demonstrate that the artificial neural models yielded better predictive performance compared to traditional regression algorithms for the targeted outputs under the experimental conditions employed. However, the exceptionally high *R*^2^ metrics observed for the ANN models should be considered in conjunction with the validation methodology and test set configuration to ensure robustness and avoid overfitting.

**Table 11:** ANN performance results.

Model	Testing *R*	Testing *R*^2^	Testing MSE	Testing RMSE
EC	0.9823	0.9649	0.20859	0.45672mS/cm
Viscosity	0.9893	0.9787	0.027348	0.16537 mPa·s
TBC	0.9965	0.9931	5,907,267.03	2,430.49 CFU/mL
ToFC	0.9964	0.9927	33,782.43	183.80 CFU/mL

**Table 12:** Performance comparison of classical regression techniques and ANN approaches.

Response	Conventional multiple linear regression *R*^2^	Regression RMSE	Regression MAE	ANN test *R*^2^	ANN test RMSE
EC	0.9740	0.3571	0.2764	0.9649	0.45672
Viscosity	0.9458	0.2253	0.1680	0.9787	0.16537
TBC	0.9230	8,222.38	7,035.26	0.9931	2,430.49
ToFC	0.9344	393.10	330.00	0.9927	183.80

Among the anticipated results, TBC demonstrated the highest correlation between actual and forecasted values, followed by ToFC and viscosity, whereas EC showed relatively lower predictive accuracy. Nevertheless, these outcomes should be considered in conjunction with the validation methodology and possible constraints on broader applicability. The supplementary ANN files encompass MATLAB-generated execution functions for standalone prediction models. These functions offer a deterministic procedure for inputting the 11 model features (apart from the EC) and generating a predicted output. The deployment mechanisms thus illustrate the practical implementability of the trained models; however, they should be considered experimental prototypes rather than fully validated real-time prediction systems.

### Limitation of the study

This study does not establish definitive health benefits in humans nor demonstrate the relative safety of natural additives compared to synthetic preservatives based on toxicology or clinical data. Further toxicology testing, safety validation, sensory profiling, and extended storage investigations are required prior to making health-related or marketing claims. Also, The microbiological evaluation was limited to quantification of total cultivable bacteria and fungi and does not offer insights into microbial taxonomy, community structure, or targeted pathogen suppression. Future investigations should utilize both culture-based identification and molecular diagnostics, including 16S rRNA and ITS gene sequencing, in conjunction with focused detection of key foodborne pathogens and spoilage microorganisms. While the current findings indicate enhanced antioxidant activity, the results do not confirm synergistic interactions. Because minimum inhibitory concentration (MIC) determinations and checkerboard or FICI assays were not performed, the observed interaction indices cannot be definitively ascribed to synergy. The amount of residual ethanol present in the extracts was not assessed in this research; therefore calculated extraction efficiencies or residual ethanol levels are not available. Subsequent work should determine its precise level through suitable analytical methods.

Additionally, since the investigation was performed under standardized laboratory storage parameters and lacked formal sensory assessment, the observed preservation efficacy should not be construed as indicative of consumer acceptance or commercial shelf stability. Additional studies under controlled storage conditions, including validated sensory assessments, are necessary to establish the product’s practical shelf life and its acceptability to consumers. A drawback of the current neural network assessment is that model efficacy was measured solely on the internal training, validation, and test subsets of the original dataset, rather than on an externally sourced, independent dataset. The dataset was divided stochastically employing MATLAB’s default data-splitting method into 70: 15:15. Since the random division was performed at the sample level, multiple instances from the same treatment–storage-time condition might be distributed across different sets. This was considered a potential avenue for information disclosure and a restriction inherent in the existing neural network validation framework. Therefore, the deployment procedures exhibit computational reproducibility but do not alone guarantee generalization or predictive accuracy on unseen watermelon juice samples, formulations, or processing conditions. Future implementation of the existing modeling architecture should seek to expand validation coverage and assess the model’s predictive performance across independently generated datasets.

## Conclusion

This experimental investigation was carried out to evaluate the possibility of using bio-based additives as alternatives to artificial food additives. Watermelon juice samples treated with both synthetic and sustainable additives, were stored at a controlled storage conditions (20 ± 2 °C), for duration of 10 days, and their engineering, nutritional, antioxidant assays, phytochemicals, and microbial dynamics were evaluated by following standard procedures. The results revealed that the treatments had a significant impact on all the parameters evaluated in this study. Notably, the natural additives enhanced the thermo-rheological and electrical properties, as well as inhibited their deterioration during the storage period. Furthermore, the findings indicated that the multi-component treatments (T11 to T16) displayed potential for better antimicrobial mechanisms, compared to single-component treatments. Additionally, the derived NPBI, MACI, MIE, and SI parameters offered a benchmarking framework for assessing treatment efficacy and comparatively robust outcome of T13, when compared to other treatments, including the positive control. Among the tested formulations, T13 tends to exhibit better preservation efficacy, characterized by reduced viable bacterial and fungal populations, as well as enhanced antioxidant and engineering properties under the experimental conditions. The enhanced efficacy noted in the T13 unit, is likely resulted from the combined effects of a heterogeneous array of phytochemicals, elevated phytochemicals, the pH-lowering action of the lime juice, honey hyperosmotic properties, and potential cooperative interactions among the sustainable additives. Therefore, the observed effect cannot be solely ascribed to the particular additives present in T13.

Additionally, the linear regression analysis and ANN results indicated that, the rheological electrical and heat storage behaviors of the watermelon juice were stable throughout the storage period, while microbial growth was impaired during storage. This stability can be linked to the applied treatment. As seen in the findings, apart from the preservative nature of the natural additives, the modifications to the thermo-rheological and electrical properties of the watermelon juice are essential in the design of food processing and smart control systems. Linear modeling provided explainable insights into the dependencies between input conditions and system variables, while neural network-based models achieved high prediction accuracy for the targeted engineering and microbial response variables within the dataset. The ANN findings establish a proof-of-concept foundation for machine learning-based prediction of the studied juice properties. Nevertheless, independent datasets and extended operational parameters are necessary to mature these models into validated quality assurance frameworks. Specifically, the data obtained in this research, should not be regarded as validation of medical, toxicological, or public health benefits, or as definitive confirmation that botanical additives are safer than artificial additives. Additionally, the results are confined to the specific storage parameters and duration examined, and no formal sensory assessment was conducted; hence, additional studies are necessary to assess the formulations under refrigerated and industrial storage environments.

## Data Availability

The original contributions presented in the study are included in the article/Supplementary material, further inquiries can be directed to the corresponding authors.
