# Supplementary material for: Artificial neural network modeling of engineering properties and microbial stability of watermelon juice preserved with sustainable natural additives
Source: Front Microbiol. 2026 Oct 9;17:1977402. doi: 10.3389/fmicb.2026.1977402 (PMC13690713; doi:10.3389/fmicb.2026.1977402)
Supplement: Supplementary file 1 [file Supplementary_file_1.docx]

**Artificial neural network predication of biofluid engineering properties, antioxidant activity microbial dynamics, using sustainable preservatives systems**

**Supplementary tables**

Table S1: The electrical conductivity of the watermelon juice during storage (**mS/cm**)

| **Code** | **Day 2** | **Day 4** | **Day 6** | **Day 8** |
| --- | --- | --- | --- | --- |
| **NC** | 4.34±0.04 | 4.81±0.05 | 4.98±0.08 | 5.67±0.06 |
| **PC** | 7.93±0.04 | 8.71±0.04 | 9.45±0.03 | 10.54±0.04 |
| **T1** | 6.55±0.19 | 7.16±0.11 | 7.76±0.12 | 8.39±0.09 |
| **T2** | 6.43±0.07 | 6.93±0.06 | 7.55±0.08 | 8.13±0.05 |
| **T3** | 5.92±0.07 | 6.48±0.04 | 7.06±0.07 | 7.57±0.05 |
| **T4** | 6.48±0.19 | 7.22±0.30 | 7.93±0.18 | 8.57±0.17 |
| **T5** | 8.96±0.29 | 9.64±0.32 | 10.58±0.34 | 11.860.39 |
| **T6** | 7.08±0.04 | 7.71±0.09 | 8.47±0.07 | 9.24±0.10 |
| **T7** | 6.74±0.05 | 7.31±0.05 | 8.04±0.10 | 8.79±0.02 |
| **T8** | 7.43±0.04 | 8.02±0.04 | 8.79±0.04 | 9.61±0.04 |
| **T9** | 7.85±0.03 | 8.51±0.03 | 9.35±0.04 | 10.23±0.04 |
| **T10** | 7.43±0.04 | 8.02±0.07 | 8.81±0.04 | 9.63±0.08 |
| **T11** | 7.81±0.04 | 8.46±0.05 | 9.27±0.05 | 9.89±0.03 |
| **T12** | 7.42±0.05 | 7.98±0.14 | 8.84±0.05 | 9.72±0.19 |
| **T13** | 9.55±0.22 | 10.29±0.23 | 11.21±0.09 | 11.99±0.15 |
| **T14** | 7.93±0.09 | 8.56±0.08 | 9.35±0.09 | 10.29±0.13 |
| **T15** | 9.20±0.06 | 9.90±0.12 | 10.01±0.16 | 11.73±0.16 |
| **T16** | 7.69±0.05 | 8.29±0.08 | 9.05±0.09 | 9.89±0.06 |

Mean ± SD

Table S2: The electrical resistivity of the watermelon juice during storage (**Ω•m**)

| **Code** | **Day 2** | **Day 4** | **Day 6** | **Day 8** |
| --- | --- | --- | --- | --- |
| **NC** | 2.30±0.03 | 2.08±0.02 | 2.01±0.03 | 1.76±0.02 |
| **PC** | 1.26±0.01 | 1.15±0.01 | 1.06±0.01 | 0.95±0.00 |
| **T1** | 1.53±0.04 | 1.40±0.02 | 1.29±0.02 | 1.19±0.01 |
| **T2** | 1.560.01 | 1.44±0.01 | 1.33±0.01 | 1.23±0.01 |
| **T3** | 1.69±0.02 | 1.54±0.01 | 1.41±0.02 | 1.32±0.01 |
| **T4** | 1.55±0.05 | 1.39±0.06 | 1.26±0.03 | 1.17±0.02 |
| **T5** | 1.12±0.03 | 1.04±0.03 | 0.95±0.03 | 0.84±0.03 |
| **T6** | 1.41±0.01 | 1.30±0.02 | 1.18±0.01 | 1.08±0.01 |
| **T7** | 1.48±0.01 | 1.37±0.01 | 1.24±0.02 | 1.14±0.00 |
| **T8** | 1.34±0.01 | 1.25±0.01 | 1.14±0.01 | 1.04±0.00 |
| **T9** | 1.28±0.01 | 1.18±0.01 | 1.07±0.00 | 0.98±0.01 |
| **T10** | 1.34±0.01 | 1.25±0.01 | 1.14±0.01 | 1.04±0.01 |
| **T11** | 1.28±0.01 | 1.18±0.01 | 1.08±0.01 | 1.01±0.00 |
| **T12** | 1.35±0.01 | 1.25±0.03 | 1.13±0.01 | 1.03±0.02 |
| **T13** | 1.05±0.03 | 0.97±0.02 | 0.89±0.01 | 0.84±0.01 |
| **T14** | 1.26±0.01 | 1.17±0.01 | 1.07±0.01 | 0.97±0.01 |
| **T15** | 1.09±0.01 | 1.01±0.01 | 1.00±0.02 | 0.85±0.02 |
| **T16** | 1.30±0.01 | 1.21±0.02 | 1.11±0.02 | 1.01±0.01 |

Mean ± SD;

Table S3: The viscosity of the watermelon juice during storage **(mPa*s)**

| **Code** | **Day 2** | **Day 4** | **Day 6** | **Day 8** |
| --- | --- | --- | --- | --- |
| **NC** | 2.18±0.04 | 2.79±0.04 | 2.99±0.07 | 3.18±0.06 |
| **PC** | 2.80±0.04 | 3.38±0.05 | 3.53±0.03 | 3.69±0.05 |
| **T1** | 3.74±0.16 | 3.94±0.17 | 3.87±0.16 | 3.71±0.16 |
| **T2** | 3.90±0.07 | 4.14±0.05 | 4.00±0.02 | 3.86±0.06 |
| **T3** | 3.77±0.29 | 3.97±0.30 | 3.90±0.29 | 3.73±0.28 |
| **T4** | 4.00±0.09 | 4.19±0.06 | 4.14±0.09 | 3.97±0.08 |
| **T5** | 3.07±0.07 | 3.24±0.08 | 3.19±0.07 | 3.05±0.07 |
| **T6** | 5.49±0.06 | 5.85±0.07 | 5.69±0.06 | 5.44±0.06 |
| **T7** | 4.43±0.07 | 4.67±0.07 | 4.59±0.07 | 4.39±0.07 |
| **T8** | 5.35±0.15 | 5.71±0.14 | 5.54±0.15 | 5.31±0.15 |
| **T9** | 4.05±0.09 | 4.30±0.06 | 4.19±0.09 | 4.01±0.09 |
| **T10** | 4.58±0.06 | 4.82±0.06 | 4.71±0.02 | 4.54±0.06 |
| **T11** | 5.90±0.09 | 5.57±0.09 | 5.27±0.05 | 5.13±0.08 |
| **T12** | 5.61±0.06 | 5.81±0.06 | 5.71±0.06 | 5.46±0.06 |
| **T13** | 6.73±0.03 | 6.41±0.03 | 6.10±0.03 | 5.89±0.03 |
| **T14** | 6.23±0.06 | 6.07±0.06 | 5.79±0.06 | 5.63±0.06 |
| **T15** | 6.22±0.04 | 6.05±0.05 | 5.90±0.04 | 5.52±0.04 |
| **T16** | 6.37±0.03 | 6.28±0.03 | 6.14±0.03 | 5.70±0.03 |

Mean ± SD;

Table S4: The Density of the watermelon juice during storage **(kg/m^3^)**

| **Code** | **Day 2** | **Day 4** | **Day 6** | **Day 8** |
| --- | --- | --- | --- | --- |
| **NC** | 937.85±11.30 | 934.08±11.29 | 929.36±11.28 | 914.28±11.26 |
| **PC** | 953.58±11.64 | 950.71±11.64 | 947.83±11.64 | 938.26±11.62 |
| **T1** | 963.70±11.20 | 961.86±11.20 | 959.93±11.20 | 952.20±11.19 |
| **T2** | 966.58±10.64 | 964.74±10.64 | 962.80±10.64 | 955.05±10.63 |
| **T3** | 960.63±10.64 | 958.80±10.64 | 956.87±10.64 | 949.16±10.63 |
| **T4** | 969.48±10.51 | 967.63±10.51 | 965.68±10.51 | 957.90±10.50 |
| **T5** | 955.68±10.37 | 953.86±10.37 | 951.94±10.37 | 944.27±10.36 |
| **T6** | 986.44±10.39 | 984.56±10.39 | 982.58±10.39 | 974.66±10.39 |
| **T7** | 974.51±10.54 | 972.65±10.54 | 970.70±10.54 | 962.88±10.54 |
| **T8** | 982.45±10.68 | 980.57±10.68 | 978.60±10.68 | 970.72±10.67 |
| **T9** | 968.39±10.52 | 966.54±10.52 | 964.60±10.52 | 956.83±10.52 |
| **T10** | 976.01±10.07 | 974.15±10.07 | 972.19±10.08 | 964.36±10.07 |
| **T11** | 994.66±10.27 | 994.06±10.27 | 992.07±10.27 | 988.08±10.27 |
| **T12** | 989.33±10.59 | 988.35±10.59 | 984.38±10.59 | 980.41±10.58 |
| **T13** | 1014.85±10.15 | 1013.84±10.15 | 1012.82±10.15 | 1009.77±10.14 |
| **T14** | 1000.37±13.33 | 998.66±13.33 | 996.66±13.32 | 986.63±13.29 |
| **T15** | 1006.14±12.92 | 1005.24±12.92 | 1002.32±12.91 | 1000.30±12.90 |
| **T16** | 1000.61±12.70 | 999.10±12.70 | 997.00±12.69 | 993.48±12.68 |

Mean ± SD;

Table S5: The thermal conductivity of the watermelon juice during storage **(W/m·K)**

| **Code** | **Day 2** | **Day 4** | **Day 6** | **Day 8** |
| --- | --- | --- | --- | --- |
| **NC** | 6.80E-01±0.01 | 6.75E-01±0.01 | 6.68E-01±0.01 | 6.64E-01±0.01 |
| **PC** | 6.59E-01±0.01 | 6.54E-01±0.01 | 6.48E-01±0.01 | 6.45E-01±0.01 |
| **T1** | 6.39E-01±0.01 | 6.36E-01±0.01 | 6.34E-01±0.01 | 6.29E-01±0.01 |
| **T2** | 6.36E-01±0.01 | 6.33E-01±0.01 | 6.30E-01±0.01 | 6.27E-01±0.01 |
| **T3** | 6.49E-01±0.01 | 6.47E-01±0.01 | 6.43E-01±0.01 | 6.40E-01±0.01 |
| **T4** | 6.39E-01±0.01 | 6.36E-01±0.01 | 6.37E-01±0.01 | 6.29E-01±0.01 |
| **T5** | 6.63E-01±0.01 | 6.59E-01±0.01 | 6.57E-01±0.01 | 6.53E-01±0.01 |
| **T6** | 6.09E-01±0.01 | 6.06E-01±0.01 | 6.05E-01±0.00 | 5.99E-01±0.01 |
| **T7** | 6.25E-01±0.01 | 6.22E-01±0.01 | 6.20E-01±0.01 | 6.17E-01±0.01 |
| **T8** | 6.09E-01±0.01 | 6.06E-01±0.01 | 6.04E-01±0.01 | 5.99E-01±0.01 |
| **T9** | 6.39E-01±0.01 | 6.36E-01±0.01 | 6.33E-01±0.01 | 6.29E-01±0.01 |
| **T10** | 6.33E-01±0.01 | 6.29E-01±0.01 | 6.27E-01±0.01 | 6.22E-01±0.01 |
| **T11** | 6.06E-01±0.01 | 6.03E-01±0.01 | 6.00E-01±0.01 | 5.99E-01±0.01 |
| **T12** | 6.14E-01±0.01 | 6.10E-01±0.01 | 6.07E-01±0.01 | 6.04E-01±0.00 |
| **T13** | 5.86E-01±0.01 | 5.83E-01±0.01 | 5.81E-01±0.01 | 5.80E-01±0.01 |
| **T14** | 5.93E-01±0.02 | 5.89E-01±0.02 | 5.87E-01±0.02 | 5.85E-01±0.02 |
| **T15** | 5.95E-01±0.01 | 5.93E-01±0.01 | 5.89E-01±0.01 | 5.87E-01±0.01 |
| **T16** | 5.98E-01±0.01 | 5.94E-01±0.01 | 5.91E-01±0.01 | 5.90E-01±0.01 |

Mean ± SD;

Table S6: The specific heat capacity of the watermelon juice during storage **(kJ/kg·K)**

| **Code** | **Day 2** | **Day 4** | **Day 6** | **Day 8** |
| --- | --- | --- | --- | --- |
| **NC** | 4.62±0.03 | 4.60±0.03 | 4.59±0.04 | 4.58±0.04 |
| **PC** | 4.33±0.11 | 4.33±0.11 | 4.32±0.11 | 4.32±0.10 |
| **T1** | 4.35±0.03 | 4.35±0.03 | 4.33±0.03 | 4.32±0.03 |
| **T2** | 4.28±0.06 | 4.27±0.06 | 4.27±0.06 | 4.27±0.06 |
| **T3** | 4.34±0.12 | 4.34±0.10 | 4.32±0.12 | 4.32±0.12 |
| **T4** | 4.27±0.04 | 4.25±0.05 | 4.25±0.04 | 4.25±0.04 |
| **T5** | 4.48±0.08 | 4.47±0.07 | 4.46±0.08 | 4.46±0.08 |
| **T6** | 4.07±0.03 | 4.05±0.04 | 4.05±0.05 | 4.03±0.05 |
| **T7** | 4.23±0.03 | 4.22±0.03 | 4.21±0.03 | 4.21±0.02 |
| **T8** | 4.19±0.06 | 4.18±0.04 | 4.16±0.05 | 4.16±0.05 |
| **T9** | 4.30±0.02 | 4.29±0.02 | 4.28±0.02 | 4.28±0.02 |
| **T10** | 4.20±0.02 | 4.20±0.03 | 4.19±0.03 | 4.18±0.03 |
| **T11** | 4.12±0.04 | 4.11±0.04 | 4.12±0.04 | 4.09±0.03 |
| **T12** | 4.15±0.04 | 4.13±0.04 | 4.12±0.04 | 4.12±0.04 |
| **T13** | 3.87±0.08 | 3.86±0.08 | 3.85±0.09 | 3.84±0.07 |
| **T14** | 4.05±0.08 | 4.04±0.07 | 4.02±0.07 | 4.02±0.07 |
| **T15** | 4.00±0.06 | 3.99±0.06 | 4.00±0.07 | 3.98±0.06 |
| **T16** | 4.06±0.05 | 4.06±0.05 | 4.04±0.06 | 4.04±0.06 |

Mean ± SD;

Table S7: The shear stress of the watermelon juice during storage **(Pa)**

| **Code** | **Day 2** | **Day 4** | **Day 6** | **Day 8** |
| --- | --- | --- | --- | --- |
| **NC** | 0.61±0.02 | 0.65±0.02 | 0.72±0.02 | 0.73±0.02 |
| **PC** | 0.96±0.01 | 1.02±0.02 | 1.08±0.03 | 1.12±0.02 |
| **T1** | 1.32±0.02 | 1.41±0.02 | 1.50±0.03 | 1.54±0.02 |
| **T2** | 1.45±0.01 | 1.53±0.01 | 1.63±0.02 | 1.68±0.01 |
| **T3** | 1.18±0.04 | 1.26±0.03 | 1.32±0.02 | 1.37±0.04 |
| **T4** | 1.50±0.03 | 1.58±0.03 | 1.69±0.04 | 1.75±0.04 |
| **T5** | 0.80±0.06 | 0.78±0.07 | 0.75±0.06 | 0.72±0.06 |
| **T6** | 2.18±0.04 | 2.12±0.03 | 2.00±0.04 | 1.96±0.03 |
| **T7** | 1.56±0.06 | 1.50±0.06 | 1.45±0.04 | 1.39±0.03 |
| **T8** | 1.95±0.07 | 1.88±0.07 | 1.80±0.05 | 1.78±0.04 |
| **T9** | 1.37±0.03 | 1.32±0.01 | 1.27±0.03 | 1.23±0.02 |
| **T10** | 1.69±0.03 | 1.64±0.03 | 1.57±0.02 | 1.53±0.05 |
| **T11** | 2.23±0.50 | 2.43±0.02 | 2.28±0.05 | 2.26±0.03 |
| **T12** | 2.27±0.03 | 2.19±0.03 | 2.11±0.03 | 2.05±0.01 |
| **T13** | 3.39±0.03 | 3.27±0.01 | 3.10±0.02 | 3.01±0.03 |
| **T14** | 2.66±0.03 | 2.62±0.04 | 2.45±0.02 | 2.38±0.03 |
| **T15** | 2.81±0.05 | 2.70±0.05 | 2.58±0.04 | 2.52±0.01 |
| **T16** | 2.69±0.04 | 2.61±0.07 | 2.49±0.03 | 2.44±0.06 |

Mean ± SD;

Table S8: The dielectric constant of the watermelon juice during storage

| **Code** | **Day 2** | **Day 4** | **Day 6** | **Day 8** |
| --- | --- | --- | --- | --- |
| **NC** | 84.28±2.34 | 83.03±2.35 | 80.95±1.75 | 79.97±1.28 |
| **PC** | 76.94±0.51 | 75.99±0.48 | 74.61±0.39 | 74.28±2.07 |
| **T1** | 74.49±0.24 | 73.73±0.23 | 72.98±0.23 | 71.85±0.22 |
| **T2** | 73.39±0.69 | 72.68±0.74 | 71.59±0.45 | 70.79±0.67 |
| **T3** | 75.34±0.83 | 74.57±0.83 | 73.21±1.85 | 73.00±0.32 |
| **T4** | 72.77±0.53 | 72.00±0.55 | 71.30±0.52 | 70.19±0.51 |
| **T5** | 78.87±0.88 | 77.89±0.74 | 76.92±0.80 | 76.49±0.53 |
| **T6** | 64.47±0.80 | 63.80±0.80 | 63.16±0.79 | 62.44±0.50 |
| **T7** | 69.81±1.77 | 68.82±1.36 | 68.39±1.73 | 67.26±1.80 |
| **T8** | 67.04±1.33 | 66.33±1.27 | 65.64±1.26 | 65.31±0.74 |
| **T9** | 71.62±1.98 | 70.66±1.65 | 69.69±1.35 | 69.05±1.86 |
| **T10** | 61.92±2.37 | 61.29±2.24 | 66.80±0.66 | 65.92±0.76 |
| **T11** | 59.22±0.79 | 59.10±0.78 | 58.50±0.79 | 57.47±0.55 |
| **T12** | 65.60±1.10 | 65.77±1.14 | 64.25±1.11 | 63.26±1.09 |
| **T13** | 59.26±1.03 | 59.17±1.07 | 58.93±0.87 | 58.59±0.98 |
| **T14** | 63.07±1.53 | 63.19±1.53 | 61.79±1.49 | 60.83±1.48 |
| **T15** | 61.23±1.12 | 61.23±1.12 | 60.85±1.25 | 60.06±0.98 |
| **T16** | 62.64±0.97 | 62.75±1.00 | 61.76±0.99 | 60.90±0.74 |

Mean ± SD;

Figure S1: ANN Model Training Set-up in Mathlab software for Electrical Conductivity

Figure S2: Model training progress for Electrical Conductivity

Figure S3: Model Train Results in Training, Validation and Testing for Electrical Conductivity

Figure S4: Model performance during Training, Validation and Testing for Electrical Conductivity

Figure S5: Model State (Status) during Training, Validation and Testing for Electrical Conductivity

Figure S6: Model Error histogram during Training, Validation and Testing for Electrical Conductivity

APPENDIX 1: Code for Electrical Conductivity model development

```
% Solve an Input-Output Fitting problem with a Neural Network
% Script generated by Neural Fitting app
% Created 24-Sep-2026 21:39:22
%
% This script assumes these variables are defined:
%
%   data - input data.
%   data_1 - target data.

x = data';
t = data_1';

% Choose a Training Function
% For a list of all training functions type: help nntrain
% 'trainlm' is usually fastest.
% 'trainbr' takes longer but may be better for challenging problems.
% 'trainscg' uses less memory. Suitable in low memory situations.
trainFcn = 'trainlm';  % Levenberg-Marquardt backpropagation.

% Create a Fitting Network
hiddenLayerSize = 10;
net = fitnet(hiddenLayerSize,trainFcn);

% Setup Division of Data for Training, Validation, Testing
net.divideParam.trainRatio = 70/100;
net.divideParam.valRatio = 15/100;
net.divideParam.testRatio = 15/100;

% Train the Network
[net,tr] = train(net,x,t);

% Test the Network
y = net(x);
e = gsubtract(t,y);
performance = perform(net,t,y)

% View the Network
view(net)

% Plots
% Uncomment these lines to enable various plots.
%figure, plotperform(tr)
%figure, plottrainstate(tr)
%figure, ploterrhist(e)
%figure, plotregression(t,y)
%figure, plotfit(net,x,t)
```

APPENDIX 2: Deployment Code for Electrical Conductivity Model Prediction

```
function [Y,Xf,Af] = myNeuralNetworkFunction(X,~,~)
%MYNEURALNETWORKFUNCTION neural network simulation function.
%
% Auto-generated by MATLAB, 24-Sep-2026 21:42:06.
%
% [Y] = myNeuralNetworkFunction(X,~,~) takes these arguments:
%
%   X = 1xTS cell, 1 inputs over TS timesteps
%   Each X{1,ts} = Qx10 matrix, input #1 at timestep ts.
%
% and returns:
%   Y = 1xTS cell of 1 outputs over TS timesteps.
%   Each Y{1,ts} = Qx1 matrix, output #1 at timestep ts.
%
% where Q is number of samples (or series) and TS is the number of timesteps.

%#ok<*RPMT0>

% ===== NEURAL NETWORK CONSTANTS =====

% Input 1
x1_step1.xoffset = [1;1;1.92;894.4;0.565;3.75;0.58;47.16;5800;450];
x1_step1.gain = [0.4;0.117647058823529;0.41407867494824;0.0164473684210526;16;2.1978021978022;0.671140939597315;0.0488997555012225;6.77048070412999e-06;0.000138026224982747];
x1_step1.ymin = -1;

% Layer 1
b1 = [-2.286426253188823754;-2.3407840673572701817;2.5936861281530854129;-1.320366622876930407;0.40399051343964709204;0.8505724512730730158;-0.88947288116524569901;0.34894321152712337586;1.4794718968203852683;-0.0035135165918245142569];
IW1_1 = [-1.7805622184618601356 -0.62445477256893977547 1.7275605199095425135 -1.7964778964150533991 0.86960065815687337398 -0.088963269730588587225 -0.92064672769946276354 0.75344266525364156362 0.486242563648574877 0.5914659292960355863;-0.027827081124631072861 3.5215248306522184407 -1.9522031554299403844 -1.5430417782105967373 0.51646237764790825508 -0.1935464012571527781 -1.8117531038455871872 0.51591610920429864517 -0.4405834150177140307 1.1553098105262622486;0.26141956097831825501 0.022341450100257440048 -0.037111141744335419956 1.7923389848815303171 -0.14983316301269275495 -0.54236754717997925912 1.0056945039878322223 1.0142774115459249895 1.1518519166672116505 0.77958160797385411822;0.83966696175007360381 -0.23490609500792233866 1.4836516532882011443 -2.2725068665637513377 0.15625569272517653152 0.25434195007912990816 0.42639411429430462697 -0.53094869573373215665 -0.85195889002875280216 0.33267171368004228027;-0.25850840410076969533 0.35995501639791527282 1.5226296722512921988 -1.6020897993693790085 -0.82318887888247149753 0.7408889047625295543 -0.65300162011498974479 0.9258675512279805897 0.42494214829488108354 -0.29774599514065480665;-1.3311522696465301063 -0.23316491382058202042 1.4651542984815597581 -1.4857292983386145568 -0.0061038261850802355557 -1.2270240579700488759 -0.66788698094523213378 0.81879308725643118461 0.26715715109570242758 -1.5145905285518932448;0.27156814276074381365 0.36575791578617616873 -1.2600066527040496567 -0.63123198500529087429 0.70389441212256187441 1.1995261188452557288 0.62513507679542346107 -2.1849281971731895347 -1.7516801030591286903 1.0083707150099383565;0.70905497821388308033 5.0557553533802481738 -0.2517768664741812934 0.64898647245010010476 0.25494736164452680427 1.4533746512931626782 1.7533180176212195267 1.7892171502586584619 -0.27362042525052432396 -0.45104043447520447696;0.13311543497759797949 0.74656047794860058708 -0.80396549688137530776 0.25419055318283639844 0.62056430538844098432 -0.20163060567800339395 -1.3970803455079428002 0.78615907141981022921 -0.54573994442357187662 -1.4650816863211759689;-2.3425308567367415513 0.34895326582526559056 -0.05823640310584661528 -1.3966890314385931315 -0.098058443355567126432 -0.78744597725276122002 -0.6924424132404161103 0.37873310336826193634 1.3578072949767425737 0.32698694042802356474];

% Layer 2
b2 = 0.11776189538510978272;
LW2_1 = [-0.82230660135241606667 1.1332339855907551662 -0.95530868646631272867 0.67605709128851487755 -0.45515054161398305066 -0.52504510527188352764 -0.7547948076425150532 0.19231371564390012385 1.0999791868025501351 -0.34590543252768907179];

% Output 1
y1_step1.ymin = -1;
y1_step1.gain = 0.177619893428064;
y1_step1.xoffset = 3.87;

% ===== SIMULATION ========

% Format Input Arguments
isCellX = iscell(X);
if ~isCellX
    X = {X};
end

% Dimensions
TS = size(X,2); % timesteps
if ~isempty(X)
    Q = size(X{1},1); % samples/series
else
    Q = 0;
end

% Allocate Outputs
Y = cell(1,TS);

% Time loop
for ts=1:TS

    % Input 1
    X{1,ts} = X{1,ts}';
    Xp1 = mapminmax_apply(X{1,ts},x1_step1);

    % Layer 1
    a1 = tansig_apply(repmat(b1,1,Q) + IW1_1*Xp1);

    % Layer 2
    a2 = repmat(b2,1,Q) + LW2_1*a1;

    % Output 1
    Y{1,ts} = mapminmax_reverse(a2,y1_step1);
    Y{1,ts} = Y{1,ts}';
end

% Final Delay States
Xf = cell(1,0);
Af = cell(2,0);

% Format Output Arguments
if ~isCellX
    Y = cell2mat(Y);
end
end

% ===== MODULE FUNCTIONS ========

% Map Minimum and Maximum Input Processing Function
function y = mapminmax_apply(x,settings)
y = bsxfun(@minus,x,settings.xoffset);
y = bsxfun(@times,y,settings.gain);
y = bsxfun(@plus,y,settings.ymin);
end

% Sigmoid Symmetric Transfer Function
function a = tansig_apply(n,~)
a = 2 ./ (1 + exp(-2*n)) - 1;
end

% Map Minimum and Maximum Output Reverse-Processing Function
function x = mapminmax_reverse(y,settings)
x = bsxfun(@minus,y,settings.ymin);
x = bsxfun(@rdivide,x,settings.gain);
x = bsxfun(@plus,x,settings.xoffset);
end
```

Figure S7: ANN Model Training Set-up in Mathlab software for Viscosity

Figure S8: Model training progress for Viscosity

Figure S9: Model Train Results in Training, Validation and Testing for Viscosity

Figure S10: Model State (Status) during Training, Validation and Testing for Viscosity

Figure S11: Model Error histogram during Training, Validation and Testing for Viscosity

APPENDIX 3: Code for Viscosity model development

```
% Solve an Input-Output Fitting problem with a Neural Network
% Script generated by Neural Fitting app
% Created 30-May-2026 11:07:02
%
% This script assumes these variables are defined:
%
%   data_1 - input data.
%   data_2 - target data.

x = data_1';
t = data_2';

% Choose a Training Function
% For a list of all training functions type: help nntrain
% 'trainlm' is usually fastest.
% 'trainbr' takes longer but may be better for challenging problems.
% 'trainscg' uses less memory. Suitable in low memory situations.
trainFcn = 'trainlm';  % Levenberg-Marquardt backpropagation.

% Create a Fitting Network
hiddenLayerSize = 10;
net = fitnet(hiddenLayerSize,trainFcn);

% Setup Division of Data for Training, Validation, Testing
net.divideParam.trainRatio = 70/100;
net.divideParam.valRatio = 15/100;
net.divideParam.testRatio = 15/100;

% Train the Network
[net,tr] = train(net,x,t);

% Test the Network
y = net(x);
e = gsubtract(t,y);
performance = perform(net,t,y)

% View the Network
view(net)

% Plots
% Uncomment these lines to enable various plots.
%figure, plotperform(tr)
%figure, plottrainstate(tr)
%figure, ploterrhist(e)
%figure, plotregression(t,y)
%figure, plotfit(net,x,t)
```

APPENDIX 4: Deployment Code for Electrical Conductivity Model Prediction

```
function [Y,Xf,Af] = myNeuralNetworkFunction(X,~,~)
%MYNEURALNETWORKFUNCTION neural network simulation function.
%
% Auto-generated by MATLAB, 30-May-2026 11:08:34.
%
% [Y] = myNeuralNetworkFunction(X,~,~) takes these arguments:
%
%   X = 1xTS cell, 1 inputs over TS timesteps
%   Each X{1,ts} = Qx11 matrix, input #1 at timestep ts.
%
% and returns:
%   Y = 1xTS cell of 1 outputs over TS timesteps.
%   Each Y{1,ts} = Qx1 matrix, output #1 at timestep ts.
%
% where Q is number of samples (or series) and TS is the number of timesteps.

%#ok<*RPMT0>

% ===== NEURAL NETWORK CONSTANTS =====

% Input 1
x1_step1.xoffset = [1;1;3.87;0.66;894.4;0.565;3.75;0.58;47.16;5800;450];
x1_step1.gain = [0.4;0.117647058823529;0.177619893428064;1.04166666666667;0.0164473684210526;16;2.1978021978022;0.671140939597315;0.0488997555012225;6.77048070412999e-06;0.000138026224982747];
x1_step1.ymin = -1;

% Layer 1
b1 = [-1.3374265035303340987;-0.31658086285216757716;0.49464287457100264422;-0.81218404115342890393;0.24428676815433328362;0.41414003925046488641;-0.26193622513462638812;-0.94306622782170068664;-1.2558810058823981493;-1.4009572370695484445];
IW1_1 = [0.58368336609424209538 0.37978728045218618226 0.44184662281000991468 -0.53379485882672017549 1.1560817643004650535 -0.32971490484385218567 0.27994919417174568554 0.55434487053043279836 0.56594808743093216741 -0.66029664833714929451 0.010977809352690305611;1.0730346506690611186 -0.64643691518670565177 -0.9342944681341729396 0.93661060713174626446 -0.85704340542976531481 0.95617944927091358398 -0.23915450302147403527 -0.58756268154143287408 1.1502569764508250305 -0.36288314878986682732 -0.10330773469821638577;-0.58275818821503133638 1.0544050254560888291 0.96136354995226236397 -0.11430563456034412151 -0.28900951084232340271 0.85755936481337402544 -0.51764249110316229441 -0.43158446658581001154 1.4522098104815945874 0.48651277925756741816 0.096562819250442313845;0.91909035871673461227 0.051560554874692722527 -0.24737226339286252919 -0.3988563162832327591 0.61255956805762823425 -0.69701395781706143584 0.29666444982969392141 0.091884399601864064078 -1.51736792529917075 0.11974652524653672803 1.0887154582639915201;0.20844723473493043109 -1.1512041341230125902 0.21761414191326677869 0.60518904881039414789 0.19543228315676186568 -0.014011764712206026262 0.21486613248652283725 -0.87437351389635342702 -0.064916816615458922124 -0.40742338635548042136 -0.75075238422190482268;0.51454162196439612309 -0.8085174448694643079 -0.68645598931415297006 0.040013739683620923315 -0.53181447392699565668 0.17756103383544788454 0.13502189541549006191 0.27732912797576514397 1.2994526275121314196 0.2548061223678600995 -0.11480369333854308078;-0.81704515571596902923 0.20075511680184726648 0.55638696484396288344 -0.31210161110516015937 0.39556476182163502475 -0.29872965395859585325 0.059602782513293685318 -0.42500865313569641257 -0.92318796212581155647 -0.55070814454720573572 -0.5407118138670108376;0.60398601031428944541 -0.35668782734429793635 -1.9347031587184859713 1.6464621825458212179 1.1583945526455983188 1.0832768930800562579 -0.62773199564919934801 -0.67589393139753317641 0.84661964865242700373 0.44773092266636860259 0.91335790679390138624;0.0085213574722423583024 -0.35847887966923741576 -0.6975488526384157062 0.44518782007925405964 -0.99584378894015279648 -0.10060642042444192801 0.038964176245531882692 0.39717877814420998739 -0.30835737622678288661 -0.45771724582639422563 -0.12148316635067257785;1.000041530545052515 0.20222176304183553386 -0.19186353538592784695 -0.83545279669895178287 0.15743763287710249577 0.1047746906753098256 0.085401973193719560307 0.53926836943450429107 0.31485814949428941878 -2.137141544278027272 0.35798200722014095199];

% Layer 2
b2 = -0.66383304193912107127;
LW2_1 = [-0.44961459065464992602 -0.68881977061664423356 -0.46363202864519192925 -0.33320495348791767665 -0.70642778006709661121 0.58357379447415713702 0.37624528265044060449 0.78806177513568886894 -1.1491535603586684466 0.60265981888093977936];

% Output 1
y1_step1.ymin = -1;
y1_step1.gain = 0.41407867494824;
y1_step1.xoffset = 1.92;

% ===== SIMULATION ========

% Format Input Arguments
isCellX = iscell(X);
if ~isCellX
    X = {X};
end

% Dimensions
TS = size(X,2); % timesteps
if ~isempty(X)
    Q = size(X{1},1); % samples/series
else
    Q = 0;
end

% Allocate Outputs
Y = cell(1,TS);

% Time loop
for ts=1:TS

    % Input 1
    X{1,ts} = X{1,ts}';
    Xp1 = mapminmax_apply(X{1,ts},x1_step1);

    % Layer 1
    a1 = tansig_apply(repmat(b1,1,Q) + IW1_1*Xp1);

    % Layer 2
    a2 = repmat(b2,1,Q) + LW2_1*a1;

    % Output 1
    Y{1,ts} = mapminmax_reverse(a2,y1_step1);
    Y{1,ts} = Y{1,ts}';
end

% Final Delay States
Xf = cell(1,0);
Af = cell(2,0);

% Format Output Arguments
if ~isCellX
    Y = cell2mat(Y);
end
end

% ===== MODULE FUNCTIONS ========

% Map Minimum and Maximum Input Processing Function
function y = mapminmax_apply(x,settings)
y = bsxfun(@minus,x,settings.xoffset);
y = bsxfun(@times,y,settings.gain);
y = bsxfun(@plus,y,settings.ymin);
end

% Sigmoid Symmetric Transfer Function
function a = tansig_apply(n,~)
a = 2 ./ (1 + exp(-2*n)) - 1;
end

% Map Minimum and Maximum Output Reverse-Processing Function
function x = mapminmax_reverse(y,settings)
x = bsxfun(@minus,y,settings.ymin);
x = bsxfun(@rdivide,x,settings.gain);
x = bsxfun(@plus,x,settings.xoffset);
end
```

Figure S12: ANN Model Training Set-up in Mathlab software for Total Bacterial Count

Figure S13: Model training progress for Total Bacterial Count

Figure S14: Model Train Results in Training, Validation and Testing for Total Bacterial Count

Figure S15: Model State (Status) during Training, Validation and Testing for Total Bacterial Count

APPENDIX 5: Code for Total Bacterial Count model development

```
% Solve an Input-Output Fitting problem with a Neural Network
% Script generated by Neural Fitting app
% Created 30-May-2026 11:24:32
%
% This script assumes these variables are defined:
%
%   data_3 - input data.
%   data_4 - target data.

x = data_3';
t = data_4';

% Choose a Training Function
% For a list of all training functions type: help nntrain
% 'trainlm' is usually fastest.
% 'trainbr' takes longer but may be better for challenging problems.
% 'trainscg' uses less memory. Suitable in low memory situations.
trainFcn = 'trainlm';  % Levenberg-Marquardt backpropagation.

% Create a Fitting Network
hiddenLayerSize = 10;
net = fitnet(hiddenLayerSize,trainFcn);

% Setup Division of Data for Training, Validation, Testing
net.divideParam.trainRatio = 70/100;
net.divideParam.valRatio = 15/100;
net.divideParam.testRatio = 15/100;

% Train the Network
[net,tr] = train(net,x,t);

% Test the Network
y = net(x);
e = gsubtract(t,y);
performance = perform(net,t,y)

% View the Network
view(net)

% Plots
% Uncomment these lines to enable various plots.
%figure, plotperform(tr)
%figure, plottrainstate(tr)
%figure, ploterrhist(e)
%figure, plotregression(t,y)
%figure, plotfit(net,x,t)
```

APPENDIX 6: Deployment Code for Total Bacterial Count Model Prediction

```
function [Y,Xf,Af] = myNeuralNetworkFunction(X,~,~)
%MYNEURALNETWORKFUNCTION neural network simulation function.
%
% Auto-generated by MATLAB, 30-May-2026 11:26:29.
%
% [Y] = myNeuralNetworkFunction(X,~,~) takes these arguments:
%
%   X = 1xTS cell, 1 inputs over TS timesteps
%   Each X{1,ts} = Qx11 matrix, input #1 at timestep ts.
%
% and returns:
%   Y = 1xTS cell of 1 outputs over TS timesteps.
%   Each Y{1,ts} = Qx1 matrix, output #1 at timestep ts.
%
% where Q is number of samples (or series) and TS is the number of timesteps.

%#ok<*RPMT0>

% ===== NEURAL NETWORK CONSTANTS =====

% Input 1
x1_step1.xoffset = [1;1;3.87;0.66;1.92;894.4;0.565;3.75;0.58;47.16;450];
x1_step1.gain = [0.4;0.117647058823529;0.177619893428064;1.04166666666667;0.41407867494824;0.0164473684210526;16;2.1978021978022;0.671140939597315;0.0488997555012225;0.000138026224982747];
x1_step1.ymin = -1;

% Layer 1
b1 = [-5.2257490357698967998;6.9688925353534445506;1.2160869337590052286;5.6367842879570213555;-4.5199361570744818195;-1.3070987884077993613;-2.2179657512756780768;-2.3961344404180113798;-3.5179285091823260245;-4.7699761539974225855];
IW1_1 = [2.8690717373416658731 -0.44307054784105598388 0.41811108486703496556 3.124241058072068089 -2.4458238473413036473 8.3080163385601455417 -1.7988797858476184288 0.78602594705221773275 -3.387765053794006942 0.47270112277498965714 -4.1653803970832470327;-0.20409631440724365636 0.79101505104994807027 2.1971626781474373757 5.0202273017962699697 -1.3854510220777491725 6.0658402390454702058 -0.18673142656138080886 6.7453477851533349963 0.12549939133044177297 2.6414197842427387108 -0.58544387092604832645;-0.43094206653418404285 -0.22341636267497599899 -0.23628893788820398703 -0.83704054633381386186 -0.12384778212650365825 0.84958053448327841561 0.17806656343402146869 -0.10922410053255515727 0.0193790566670449943 0.020913554278352342042 -0.24794838384545098142;-5.7425275825033210708 -0.21616180610519777905 1.6750026444659593672 -1.1229715773529080902 1.2451042265645062557 -3.9930352317799582273 0.6005715804404885283 -0.45613246441737853587 1.8875643609649639387 0.55995626088010919119 5.4775928145887986531;2.7794650495082509067 -2.1361643999613724176 4.5680698480605403944 1.8412014821902300543 -2.3613724719450543965 14.480981784498204945 -0.41744287603619228966 1.0339068815867522577 1.1403586534060046187 -2.4531711221122147748 -6.9623099414259712603;2.0881433380317462323 -1.2879632058176651022 2.3925243583013355853 3.3694309571733036535 2.7247994372220389891 -1.2948086873883959402 5.4904047666433193342 -1.3168687520103994348 1.1800703000813776988 -0.77886679841305728189 -4.0316819551040783409;-2.4871967790349907013 -4.7978000672154301753 -7.8759491287158329698 -16.064210950342300066 -4.6146778940110699807 4.8075223973134511013 -1.2823455582456584345 -1.7140568775071911567 0.68395320918320279269 0.087920414395741913749 10.726145869122724719;1.8272511557469055532 1.1476689564236903784 1.817111546047052606 3.7589180621651383518 0.99195947244229054629 -3.9299478983259241716 -0.60414409988297468335 0.52156391752246999971 -0.023656399701487682008 -0.13988289558303707016 -5.1475828696764720505;-1.3313564177255388099 -2.054321580217413068 -0.049117658628510411922 -1.3371419042273862132 1.1858350516854503365 6.4095688349173638443 1.9785652371878927891 -2.0272360009273806547 -1.5434268425789208212 -1.694787044041868862 -0.62918359771540999237;11.695660777490711979 -1.9674207388198707847 6.1385960455037649197 15.475135099980377618 -9.7300811668386941733 33.268451641852529121 -3.3936150525150843293 -2.6621107018212275541 -12.109220218581331352 4.3129506498550584936 -4.2851312712296296681];

% Layer 2
b2 = 1.8194260309836103318;
LW2_1 = [-0.024438536466446395684 -0.26982486249634785036 -2.5806466825970968415 -0.084689494024577455233 0.034093969384768441078 0.017260487891888661588 0.013400545569764627657 -0.11090937051448958306 -0.032334604641134370717 0.010124080887976579998];

% Output 1
y1_step1.ymin = -1;
y1_step1.gain = 6.77048070412999e-06;
y1_step1.xoffset = 5800;

% ===== SIMULATION ========

% Format Input Arguments
isCellX = iscell(X);
if ~isCellX
    X = {X};
end

% Dimensions
TS = size(X,2); % timesteps
if ~isempty(X)
    Q = size(X{1},1); % samples/series
else
    Q = 0;
end

% Allocate Outputs
Y = cell(1,TS);

% Time loop
for ts=1:TS

    % Input 1
    X{1,ts} = X{1,ts}';
    Xp1 = mapminmax_apply(X{1,ts},x1_step1);

    % Layer 1
    a1 = tansig_apply(repmat(b1,1,Q) + IW1_1*Xp1);

    % Layer 2
    a2 = repmat(b2,1,Q) + LW2_1*a1;

    % Output 1
    Y{1,ts} = mapminmax_reverse(a2,y1_step1);
    Y{1,ts} = Y{1,ts}';
end

% Final Delay States
Xf = cell(1,0);
Af = cell(2,0);

% Format Output Arguments
if ~isCellX
    Y = cell2mat(Y);
end
end

% ===== MODULE FUNCTIONS ========

% Map Minimum and Maximum Input Processing Function
function y = mapminmax_apply(x,settings)
y = bsxfun(@minus,x,settings.xoffset);
y = bsxfun(@times,y,settings.gain);
y = bsxfun(@plus,y,settings.ymin);
end

% Sigmoid Symmetric Transfer Function
function a = tansig_apply(n,~)
a = 2 ./ (1 + exp(-2*n)) - 1;
end

% Map Minimum and Maximum Output Reverse-Processing Function
function x = mapminmax_reverse(y,settings)
x = bsxfun(@minus,y,settings.ymin);
x = bsxfun(@rdivide,x,settings.gain);
x = bsxfun(@plus,x,settings.xoffset);
end
```

Figure S16: ANN Model Training Set-up in Mathlab software for Total Fungal Count

Figure S17: Model training progress for Total Fungal Count

Figure S18: Model Train Results in Training, Validation and Testing for Total Fungal Count

Figure S19: Model State (Status) during Training, Validation and Testing for Total Fungal Count

Figure S20: Model Error histogram during Training, Validation and Testing for Total Fungal Count

APPENDIX 7: Code for Total Fungal Count model development

```
% Solve an Input-Output Fitting problem with a Neural Network
% Script generated by Neural Fitting app
% Created 19-Jun-2026 00:40:21
%
% This script assumes these variables are defined:
%
%   data - input data.
%   data_1 - target data.

x = data';
t = data_1';

% Choose a Training Function
% For a list of all training functions type: help nntrain
% 'trainlm' is usually fastest.
% 'trainbr' takes longer but may be better for challenging problems.
% 'trainscg' uses less memory. Suitable in low memory situations.
trainFcn = 'trainlm';  % Levenberg-Marquardt backpropagation.

% Create a Fitting Network
hiddenLayerSize = 10;
net = fitnet(hiddenLayerSize,trainFcn);

% Setup Division of Data for Training, Validation, Testing
net.divideParam.trainRatio = 70/100;
net.divideParam.valRatio = 15/100;
net.divideParam.testRatio = 15/100;

% Train the Network
[net,tr] = train(net,x,t);

% Test the Network
y = net(x);
e = gsubtract(t,y);
performance = perform(net,t,y)

% View the Network
view(net)

% Plots
% Uncomment these lines to enable various plots.
%figure, plotperform(tr)
%figure, plottrainstate(tr)
%figure, ploterrhist(e)
%figure, plotregression(t,y)
%figure, plotfit(net,x,t)
```

APPENDIX 8: Deployment Code for Total Fungal Count Model Prediction

```
function [Y,Xf,Af] = myNeuralNetworkFunction(X,~,~)
%MYNEURALNETWORKFUNCTION neural network simulation function.
%
% Auto-generated by MATLAB, 19-Jun-2026 00:38:39.
%
% [Y] = myNeuralNetworkFunction(X,~,~) takes these arguments:
%
%   X = 1xTS cell, 1 inputs over TS timesteps
%   Each X{1,ts} = Qx11 matrix, input #1 at timestep ts.
%
% and returns:
%   Y = 1xTS cell of 1 outputs over TS timesteps.
%   Each Y{1,ts} = Qx1 matrix, output #1 at timestep ts.
%
% where Q is number of samples (or series) and TS is the number of timesteps.

%#ok<*RPMT0>

% ===== NEURAL NETWORK CONSTANTS =====

% Input 1
x1_step1.xoffset = [1;1;3.87;0.66;1.92;894.4;0.565;3.75;0.58;47.16;5800];
x1_step1.gain = [0.4;0.117647058823529;0.177619893428064;1.04166666666667;0.41407867494824;0.0164473684210526;16;2.1978021978022;0.671140939597315;0.0488997555012225;6.77048070412999e-06];
x1_step1.ymin = -1;

% Layer 1
b1 = [-8.4474516591365400586;0.13369094086376204222;0.098551355248574279422;-0.10599898232546732524;0.53584846137444375191;-1.8397352809861471368;-0.92049407292642515532;1.4893366194796351376;0.70787052189004728842;14.19222649910543943];
IW1_1 = [-4.5246759364467097342 0.95933101882039151231 -2.0263570664427299484 -0.24404297270064206904 -4.6050587822085740086 2.3984114744627849092 -0.70807239652762998805 1.0377745096265145186 3.4838171736257987909 0.62769223469100643431 -4.4253246239083274816;-2.3487085231768118909 -0.44192424580962796909 2.4609272006618660988 -1.6656271091569632148 0.11290957279203307773 -0.4762226990554165873 -1.1074698835652683204 0.038044617797146844385 -3.2653500868337843599 -0.45832807528979441214 0.79018601486521222732;-0.28173470507329023693 -0.33100669413447364464 -0.3463231608835037334 -0.80207977968171439986 0.041544409779761107271 0.40678192152187370167 -0.071094317347489277559 -0.17263212489712792541 0.033606950590197769146 0.13228702867033328983 -1.0861759900967815629;1.1920616422054772787 -0.24684782514582501167 0.2067352294247252753 0.5780434771912362768 0.39741621128270016028 -0.17753411813498043426 0.24191749064308201711 -0.1657294980309896304 1.0209125037988202589 -0.012395580905317293147 -1.1156429965165957086;-2.8541278433518608715 -2.0887350498345997885 -0.25512455688614515958 -14.209623126390905412 5.331567433012557089 0.047015696700371562011 3.8821332520692841683 1.4997359791429909492 0.020262165269628651387 6.4532511739092068836 5.3267984016439351436;-9.3606174894805516118 -0.34180478262134794676 -2.2102452816941138281 -3.1574226490391961164 0.89418090914826986459 -1.1471418536169255553 -0.95854818425471499665 0.24506720266946507847 -0.78003351287705013917 -0.010277507763543199115 1.1017012256262976866;-1.1814769677072729515 0.82699048739789982054 1.4962563395971726354 2.1159849126708758504 -0.82690481678138860744 1.0716703072849818046 -0.073748096719577216351 0.80258135220341486615 -1.2565780091757685621 0.19371624956332422762 -1.1270227075189105381;0.29790981500466262588 -0.91854201553500913757 -2.3543280102095445194 -1.4533024656348221271 1.4655333347980994407 0.10263098924636836207 -0.32436525148567235677 -1.9274819140072743107 3.6847474204511065921 -1.4868018211411737628 1.1059162649752327656;0.25905810576237520149 0.048300966228463158847 4.9461221609911554253 5.7202979063924566105 1.2096918484287435547 -0.77499290214644300789 -0.12620090224039476334 -0.11874507862248245837 0.52871765969089734583 -2.0549208537385270645 -3.7613055536574586668;-3.4804498798548353378 2.0721192397109202687 5.7874425483706470885 5.9223810729208086201 0.5790399075550435315 0.34019429491097175067 -1.0086854981242792118 0.41409603129295891044 -3.6883998267345998734 -0.34296543498890252888 9.9707118015347990791];

% Layer 2
b2 = 0.80046013862690157392;
LW2_1 = [-0.059193520406558189362 0.072777756010097185979 -1.2375585713057701831 0.33246299443968924114 -0.013038378246766658963 0.060664373135016325111 0.12392126498761778242 0.049644113430314787405 -0.11233665078847154817 -0.54273045686010445987];

% Output 1
y1_step1.ymin = -1;
y1_step1.gain = 0.000138026224982747;
y1_step1.xoffset = 450;

% ===== SIMULATION ========

% Format Input Arguments
isCellX = iscell(X);
if ~isCellX
    X = {X};
end

% Dimensions
TS = size(X,2); % timesteps
if ~isempty(X)
    Q = size(X{1},1); % samples/series
else
    Q = 0;
end

% Allocate Outputs
Y = cell(1,TS);

% Time loop
for ts=1:TS

    % Input 1
    X{1,ts} = X{1,ts}';
    Xp1 = mapminmax_apply(X{1,ts},x1_step1);

    % Layer 1
    a1 = tansig_apply(repmat(b1,1,Q) + IW1_1*Xp1);

    % Layer 2
    a2 = repmat(b2,1,Q) + LW2_1*a1;

    % Output 1
    Y{1,ts} = mapminmax_reverse(a2,y1_step1);
    Y{1,ts} = Y{1,ts}';
end

% Final Delay States
Xf = cell(1,0);
Af = cell(2,0);

% Format Output Arguments
if ~isCellX
    Y = cell2mat(Y);
end
end

% ===== MODULE FUNCTIONS ========

% Map Minimum and Maximum Input Processing Function
function y = mapminmax_apply(x,settings)
y = bsxfun(@minus,x,settings.xoffset);
y = bsxfun(@times,y,settings.gain);
y = bsxfun(@plus,y,settings.ymin);
end

% Sigmoid Symmetric Transfer Function
function a = tansig_apply(n,~)
a = 2 ./ (1 + exp(-2*n)) - 1;
end

% Map Minimum and Maximum Output Reverse-Processing Function
function x = mapminmax_reverse(y,settings)
x = bsxfun(@minus,y,settings.ymin);
x = bsxfun(@rdivide,x,settings.gain);
x = bsxfun(@plus,x,settings.xoffset);
end
```
